# Circadian Activity and Clock Genes in *Pachycrepoideus vindemmiae*: Implications for Field Applications and Circadian Clock Mechanisms of Parasitoid Wasps

**DOI:** 10.3390/insects14050486

**Published:** 2023-05-22

**Authors:** Ziwen Teng, Mengran Huo, Yanan Zhou, Yuqi Zhou, Yunjie Liu, Yan Lin, Qi Zhang, Zhiqi Zhang, Fanghao Wan, Hongxu Zhou

**Affiliations:** 1 Shandong Engineering Research Center for Environment-Friendly Agricultural Pest Management, Shandong Province Laboratory for Biological Invasions and Ecological Security, China-Australia Cooperative Research Center for Crop Health and Biological Invasions, College of Plant Health & Medicine, Qingdao Agricultural University, Qingdao 266109, China; tzwbat@126.com (Z.T.); huomengran333@163.com (M.H.); 15315469602@163.com (Y.Z.); 13569718312@163.com (Y.Z.); 18263682201@163.com (Y.L.); 13327968955@163.com (Y.L.); zsscarpediem@163.com (Q.Z.); 18266095589@163.com (Z.Z.); wanfanghao@caas.cn (F.W.); 2 Agricultural Genomics Institute at Shenzhen, Chinese Academy of Agricultural Sciences, Shenzhen 518120, China

**Keywords:** *Pachycrepoideus vindemmiae*, parasitoid wasp, circadian rhythms, clock genes, field application

## Abstract

**Simple Summary:**

Insects regulate their physiology and behavior through their circadian clock in response to daily changes in the photoperiod. Parasitoid wasps are well-known biocontrol agents. Therefore, understanding the circadian activities of parasitoid adults may help improve biological control strategies. In the present study, we characterized the circadian patterns of emergence, mating, and oviposition of an ectoparasitoid wasp, *Pachycrepoideus vindemmiae*. We also identified eight clock candidate genes, most of which showed significant rhythmic expressions. These results serve as a starting point for further functional studies of the clock genes in *P. vindemmiae* as well as in other parasitoid wasps. The findings of this study also provide information that could contribute to improving biological control strategies using parasitoid wasps.

**Abstract:**

Despite the importance of circadian rhythms in insect behavior, our understanding of circadian activity and the molecular oscillatory mechanism in parasitoid wasp circadian clocks is limited. In this study, behavioral activities expected to be under the control of the endogenous circadian system were characterized in an ectoparasitoid wasp, *Pachycrepoideus vindemmiae*. Most adults exhibited emergence between late night and early morning, while mating only occurred during the daytime, with a peak at midday. Oviposition had three peaks in the early morning, late day, or early night and late night. Additionally, we identified eight putative clock genes from *P. vindemmiae*. The quantitative PCR (qPCR) results indicate that most clock genes showed significant rhythmic expressions. Our comparative analysis of clock genes in *P. vindemmiae* and 43 other parasitoid wasps revealed that none of the wasps possessed the *timeless* and *cry1* genes commonly found in some other insect species, suggesting that the circadian clock system in parasitoid wasps is distinct from that in other non-Hymenoptera insects such as *Drosophila*. Thus, this study attempted to build the first hypothetical circadian clock model for a parasitoid wasp, thus generating hypotheses and providing a platform for the future functional characterization of *P. vindemmiae* clock genes as well as those of other parasitoid wasps. Finally, these findings on *P. vindemmiae* circadian activity will aid the development of effective field release programs for biological control, which can be tested under field conditions.

## 1. Introduction

Insects regulate their physiology and behavior through their circadian clock in response to daily changes in the photoperiod. In parasitoids, the circadian system controls the timing of activities such as locomotor, emergence, mating, and oviposition activity [1]. For example, the locomotor activity of *Brachymeria intermedia* only occurs during the photophase [2], while the locomotor activity of female *Meteorus pulchricornis* wasps peaks just after light off under a photoperiod of 16:8 h (light/dark), indicating a primarily nocturnal pattern [3]. The emergence of *Cotesia kariyai* larvae occurs solely during the photophase and is not dependent on the developmental duration, and they can emerge in the new photophase under the reversed cycle [4]. The emergence of *Trichogramma brassicae* male and female adults occurs within three hours of lighting [5]. *Nasonia vitripennis* males emerge preferentially around light-on when they anticipate the light-on signal [6]. The daily oviposition rhythms of three *Drosophila* parasitoids were monitored under a light–dark cycle. *Leptopilina heterotoma* and *Asobara tabida* mainly parasitize hosts in the morning, and *L. boulardi* oviposits just before light off [7]. *Eretmocerus warrae* females oviposit throughout the 24 h period with a peak at 10–14 h into the photophase [8]. The emergence, mating, and oviposition rhythms of *Tamarixia triozae* have been recorded and analyzed, with most emergence and mating activities taking place in the morning and oviposition exclusively occurring during the daytime with a peak in the mid-morning to mid-afternoon [9].

At the molecular level, the central circadian clock has been most extensively studied in the fruit fly *Drosophila melanogaster*. The basic mechanism of the clock involves three interlocked autoregulatory transcriptional translational feedback loops consisting of a set of clock genes [10]. In the first major loop, the products of *Clock* (*Clk*) and *cycle* (*cyc*) genes form heterodimers, which activate the transcription of the *period* (*per*) and *timeless* (*tim*) during the late day to early night [11,12,13,14]. In the middle of the night, the proteins PER and TIM form heterodimers and enter the nucleus, where they suppress the transcriptional activity of the CLK/CYC complex and, thus, repress their own transcription [12,15]. Light exposure causes the *Drosophila-type cryptochrome* (CRY-d) to disrupt PER and TIM heterodimers, releasing the inhibition of transcription [1,16]. The rhythmic expression of *per* and *tim* is produced by this negative feedback. The second loop involves the genes *Clk*, *cyc*, *vrille,* and *Par domain protein 1* (*Pdp1*) [17]. During the night, CLK/CYC activates the transcription of vrille and Pdp1 through an E-box [18]. The VRILLE protein accumulates earlier than PDP1 and represses *Clk* transcription through a V/P-box. PDP1 accumulates later than VRILLE and triggers *Clk* transcription during the day, resulting in a peak of CLK during the day [10,19]. The third loop is made up of the *clockwork orange* (*cwo*) gene, a member of the orange superfamily. The protein CWO binds to the E-box, competing with CLK/CYC, to regulate the amplitude of *Clk*, *per*, and *tim* mRNA oscillations [20]. Although other insects have a basic clock mechanism similar to that of *Drosophila*, some differences have been observed [21]. For example, in *Antheraea pernyi* and *Bombyx mori*, PER does not enter the nucleus [22,23]. In the genome of hymenopteran species, *tim* is absent [24,25]. The involvement of CRY2 in the PER/TIM feedback loop has been reported in *Apis mellifera* and *Danaus plexippus* [16,24,26].

The molecular mechanisms of the circadian clock in parasitoid wasps are largely unknown. Most studies on the circadian clocks of parasitoid wasps have been limited to those of *N. vitripennis* [27]. For instance, in the heads of *N. vitripennis* females, *per* and *cry* mRNA levels were found to oscillate synchronously in L12: D12 and L16: D8 photoperiods, as well as in constant light and darkness following entrainment in the same photoperiods. The light-on signal determined the occurrence of *per* and *cry* oscillations [28]. In contrast, other clock genes, including *Clk*, *cwo*, and *Pdp1,* showed no significant rhythmic expression in the heads of *N. vitripennis* females under any photoperiods [29]. *Per* RNAi altered the expression of *cry2*, *Clk*, and *cyc*, changed the locomotor activity, and affected male courtship behavior [27,30]. Furthermore, *N. vitripennis* females injected with dsRNA of *per* were unable to produce diapause-destined eggs in response to short days, indicating that *per* plays a crucial role in the photoperiodic perception and the timing of photoperiodic diapause induction [27,29].

*Pachycrepoideus vindemmiae* (Hymenoptera: Pteromalidae), a solitary and generalist pupal wasp, can successfully parasitize a wide range of fly (Diptera) hosts [31,32,33]. The potential of *P. vindemmiae* as biological control agents against Diptera pests has been evaluated. For example, *P. vindemmiae* has been found to be one of only two parasitoid species that can successfully attack and kill *Drosophila suzukii* in the field in Europe and the Americas [34]. Despite its potential, the circadian activities of *P. vindemmiae* are not well understood, and research exploring the molecular mechanisms of its circadian clock is limited, as is the case with many other parasitoid wasps. In the present study, we characterized circadian activities under a photoperiod of 12:12 h (light/dark) and identified eight clock candidate genes of *P. vindemmiae*. The expression profiles of these genes were also determined. These results provide a basis for comparison with the circadian systems of other insect species and serve as a starting point for further functional studies of the clock genes in *P. vindemmiae* as well as in other parasitoid wasps. The findings of this study can also provide information to improve biological control strategies using parasitoid wasps.

## 2. Materials and Methods

### 2.1. Insect Rearing

The *D. melanogaster w^1118^* obtained from the Bloomington Stock Center (Indiana University, Bloomington, IL, USA) was reared on standard medium. The *P. vindemmiae* colony was kindly provided by Prof. Gongyin Ye (Zhejiang University, Hangzhou, China) and reared by parasitizing the pupae of *w^1118^* as described in [31,35,36]. Adult wasps were held in culture tubes (2.5 cm in diameter and 10 cm in height) (ASOCC507601, Sinopharm, Shanghai, China) and fed with a 20% *v*/*v* sucrose solution (A610498, Sangon Biotech, Shanghai, China). Both laboratory cultures were maintained at 25 °C and 60 ± 5% relative humidity under a photoperiod of 12:12 h of light and darkness [31].

### 2.2. The Circadian Activity of P. vindemmiae

We recorded sex-dependent emergence rhythms using a method described by Bertossa et al. [6] and Chen et al. [9]. Briefly, 15 two-day-old mated female wasps were placed in Petri dishes (10 cm in diameter) (F611004, Sangon Biotech, Shanghai, China) with 60 host pupae aged two days old and allowed to parasitize for 6 h (Zeitgeber time (ZT) 9–15; ZT0 corresponds to light on and ZT12 corresponds to light off) under a 12:12 light–dark photoperiod. A total of 16 days after oviposition, the emergence rhythms of 30 *P. vindemmiae* females (5 replicates of 6 females each) and 25 males (5 replicates of five males each) from the parasitized host pupae were observed for 3 successive days under the same photoperiod. During the scotophase, the infrared night vision system connected to a PC was used to record the observations.

For measuring circadian mating rhythm, we individually paired one-day-old virgin females with one-day-old virgin males at the beginning of the photophase under a photoperiod of 12:12 h (light/dark). The mating events of 26 pairs (5 replicates of 5 or 6 pairs each) were observed continuously throughout both the photophase and scotophase. During the scotophase, the infrared night vision system was used, as described above.

The two-day-old mated females were used for recording circadian oviposition rhythm. We paired a one-day-old virgin female with a one-day-old virgin male in a petri dish (3.5 cm in diameter) (F611201, Sangon Biotech, Shanghai, China). The male was removed immediately after a mating event was observed. Then, we obtained a mated female and fed it until this female was two days old. A single two-day-old mated female was held in the Petri dish (3.5 cm in diameter) (F611201, Sangon Biotech, Shanghai, China) with 10 host pupae aged two days old at the beginning of the photophase under a photoperiod of 12:12 h (light/dark). The oviposition rhythms of 32 mated females (5 replicates of 6 or 7 females each) were observed continuously throughout photophase and scotophase. During the scotophase, the infrared night vision system was used. Female wasps were fed on 20% *v*/*v* sucrose solution.

### 2.3. Identification of Clock Systems from Parasitoid Wasps

The *P. vindemmiae* transcriptome can be obtained from the National Center for Biotechnology Information (NCBI) Sequence Read Archive (SRA) Databases (https://www.ncbi.nlm.nih.gov/sra/PRJNA573955, accessed on 15 December 2022). The genome data of 43 other parasitoid wasps (Table S1) were obtained from InsectBase 2.0 (http://v2.insect-genome.com/, accessed on 15 December 2022). Firstly, candidate clock genes of *P. vindemmiae* and other parasitoid wasps were identified through TBLASTN searches against transcriptomes and genomes using local BLAST with an E-value cutoff of 1 × 10^−5^ using known clock protein sequences of *D. melanogaster*, *Anopheles gambiae*, *A. mellifera*, *D. plexippus*, *Tribolium castaneum*, and *Gryllus bimaculatus*. Then, candidate genes were further confirmed manually using online BLASTP versus NCBI non-redundant protein sequences without species limits (E-value: 1 × 10^−5^). 

### 2.4. Sequence Alignment and Phylogenetic Analysis

Domain analyses of CRY2, VRILLE, PDP1 and CWO proteins were conducted according to a CDD search (https://www.ncbi.nlm.nih.gov/cdd, accessed on 15 December 2022) [37]. The domains of other clock proteins were analyzed based on their corresponding references. Multiple sequence alignments of the amino acid sequences were performed using ClustalX2 [38] and then edited using GeneDoc. Phylogenetic analysis was conducted using MEGA 7 with 1000 bootstrap replicates based on the maximum likelihood method [39].

### 2.5. Quantitative Real-Time PCR (qPCR)

The mRNA levels of the clock genes from *P. vindemmiae* were measured using qPCR. One day after emergence, the heads of adult insects were collected into a TRIzol reagent (15596018, Invitrogen, Carlsbad, CA, USA) every 4 h (ZT2, 6, 10, 14, 18, 22; ZT0 corresponds to light-on and ZT12 corresponds to light-off). Total RNA was extracted from the collected heads according to the manufacturer’s protocol. The first-strand complementary DNA (cDNA) was synthesized using TransScript One-Step gDNA Removal and cDNA Synthesis SuperMix (AT311, Transgen, Beijing, China) as previously described [40]. The specific qPCR primers were designed using Primer3 Input (version 0.4.0, https://bioinfo.ut.ee/primer3-0.4.0/, accessed on 15 November 2022) (Table S2). qPCR was carried out for three biological replicates, with 20 adult heads pooled together for each replicate. The experiments were conducted on a CFX96™ Real-Time PCR Detection System (Bio-Rad, Hercules, CA, USA) using the following program: 95 °C for 30 s, followed by 40 cycles of 95 °C for 5 s, and 60 °C for 30 s. We used the 28S rRNA gene as the reference gene [31]. Relative expression levels were calculated using the comparative 2^−ΔΔCT^ method [41], following the guidelines described by Bustin et al. [42].

### 2.6. Data Analysis

The differences between the means were analyzed using one-way analysis of variance (ANOVA) and Tukey’s test with statistical significance set at *p* < 0.05 using SPSS version 22 (IBM SPSS Statistics for Windows, Version 22.0). The results of the circadian rhythm observation and qPCR were visualized using GraphPad Prism 7.0 (GraphPad, San Diego, CA, USA).

## 3. Results

### 3.1. The Circadian Activity of P. vindemmiae

**Figure 1:**
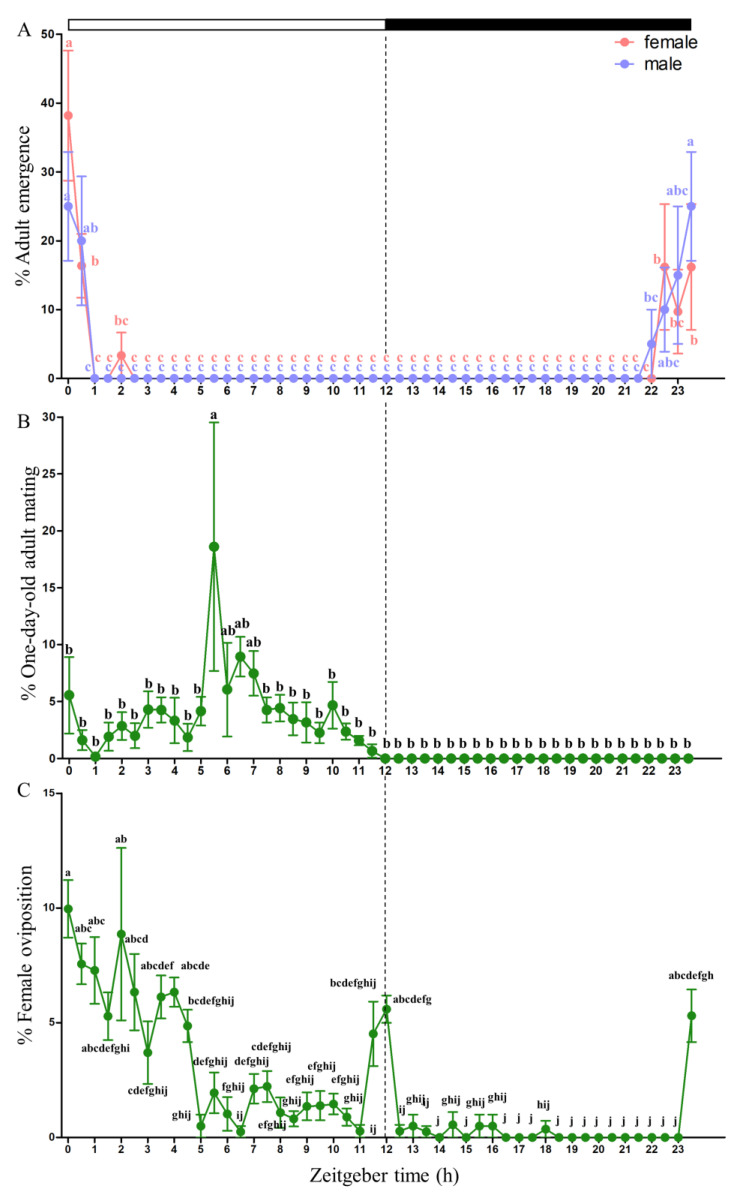
*Pachycrepoideus vindemmiae* males and females emergence(**A**), mating (**B**) and oviposition (**C**) patterns under a photoperiod of 12: 12 h (light/dark). Data are presented as the means ± standard deviation. The different letters are significantly different based on one-way analysis of variance (ANOVA) and Tukey’s test with differences considered significant at *p* < 0.05.

The emergence of both sexes was highest between the late scotophase and the early photophase and then significantly decreased. All males and females emerged from ZT 22 to ZT 2. One sex difference noted in the emergence of *P. vindemmiae* was that males started to emerge at ZT 22, half an hour earlier than females, and completed emergence at ZT 0.5, 1.5 h later than females (Figure 1A). Mating was observed only during the daytime, with the highest peak occurring at midday (Figure 1B). Oviposition had three peaks in the early morning, late day or early night, and late night. Nearly 70% of female adults oviposited within six hours into the photophase (Figure 1C).

### 3.2. Core Clock Genes in P. vindemmiae and Other Parasitoid Wasps

Eight core clock genes were identified in *P. vindemmiae*, including Clock (Pv_Clk), cycle (Pv_cyc), period (Pv_per), timeout (Pv_timout), cryptochrome2 (Pv_cry2), vrille (Pv_vrille), Par domain protein 1 (Pv_Pdp1) and clockwork orange gene (Pv_cwo) (Table 1 and Table 2). BLASTP (https://blast.ncbi.nlm.nih.gov/Blast.cgi, accessed on 5 May 2023) analysis revealed that six of the eight clock proteins exhibited the highest sequence homology with those of N. vitripennis, with the exception of Pv_VRILLE, which showed the highest sequence homology with that of Melipona quadrifasciata, and Pv_CWO, which showed the highest sequence homology with that of Colletes gigas (Table 1). tim and cry1 were not identified in all the wasps analyzed in this study, and cyc, per, cry2, and vrille were not identified in eight, one, two, and one species of these wasps, respectively. However, Clk, timeout, Pdp1, and cwo were identified in all the parasitoid wasps (Table 2 and Table S1).

### 3.3. CLK

Pv_CLK shared 79% identity (BlastP, E-value = 0) with the CLK protein from *N. vitripennis* (Table 1). Pv_CLK contains an N-terminal basic helix loop helix (bHLH) domain for binding to DNA [43]. Two PER-ARNT-SIM (PAS) domains mediating the binding to the heterodimeric partner CYC were also detected in Pv_CLK. In the region immediately carboxy-terminal to the PAS-B domain, a PAS-associated C terminal (PAC) domain was detected, which was proposed to be necessary for dimer formation (Figure S1) [44]. Phylogenetic analysis of CLKs from parasitoid wasps showed all the proteins could be divided into two groups (Figure S2). Group 1 CLKs formed three branches with CLKs from Figitidae, Ichneumonidae, and Pteromalidae. Group 2 CLKs from five families of parasitoids are separated into five clusters to be family-specific. Pv_CLK shared a much closer evolutionary relationship with CLKs from Pteromalidae in group 1.

### 3.4. CYC

CYC is also termed aryl hydrocarbon receptor nuclear translocator (ARNT) or brain and muscle ARNT-like protein (BMAL) [44,45]. Pv_CYC shared about 78% identity (BlastP, E-value = 0) with ARNT-like protein 1 isoform X2 from *N. vitripennis* (Table 1). Pv_CYC contains three highly conserved regions that are characteristic of known CYC proteins, bHLH, PAS-A, and PAS-B domains (Figure S3). The evolutionary tree showed that Pv_CYC clusters with corresponding proteins from other Pteromalidae parasitoids. The other CYCs, deriving from five families of parasitoid wasps, were separated into five clusters to be family-specific (Figure S4).

### 3.5. PER

Pv_PER had 63% sequence similarity (BlastP, E-value = 0) to PER in *N. vitripennis* (Table 1). Pv_PER contains PAS-A and PAS-B regions that mediate the binding of PER to its heterodimeric partner TIM [15]. A cytoplasmic localization domain (CLD) involved in the retention of PER in the cytoplasm [46] and nuclear localization signal (NLS) mediating nuclear entry of the PER-TIM complex [47] were also detected in Pv_PER (Figure S5). The phylogenetic tree indicated Pv_PER clusters with corresponding proteins from other Pteromalidae parasitoids. The other PERs, deriving from five families of parasitoid wasps, were separated into five clusters to be family-specific (Figure S6).

### 3.6. TIMEOUT

**Figure 2:**
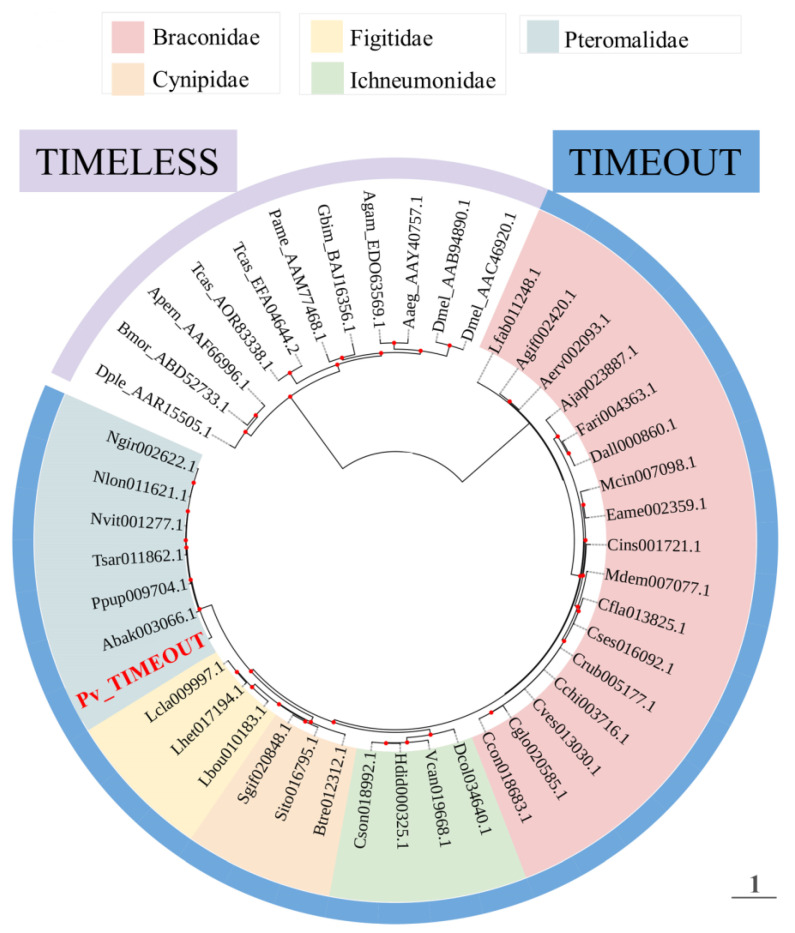
Phylogenetic relationships of TIMEOUT proteins from parasitoid wasps including *Pachycrepoideus vindemmiae* and TIMELESS proteins from *Aedes aegypti* (Aaeg), *Anopheles gambiae* (Agam), *Antheraea pernyi* (Aper), *Bombyx mori* (Bmor), *Drosophila melanogaster* (Dmel), *Danaus plexippus* (Dple), *Gryllus bimaculatus* (Gbim), *Periplaneta americana* (Pame) and *Tribolium castaneum* (Tcas). GenBank numbers of TIMELESS proteins are listed after the abbreviations of species names. For TIMEOUT proteins, tip labels show the protein names in InsectBase 2.0 and the species information is listed in Table S1. The phylogenetic tree is constructed using the maximum likelihood method. The best models is JTT+G4. Red dots at the nodes denote bootstrap values greater than 500 from 1000 trials.

Pv_TIMEOUT exhibited 89% sequence similarity (BlastP, E-value = 0) to the TIMELESS homolog from *N. vitripennis* (Table 1). Pv_TIMEOUT contains the conserved TIMELESS domain and TIMELESS-C domain as found in other species (Figure S7) [48]. A phylogenetic tree was constructed based on TIMEOUT and TIMELESS sequences for 34 parasitoid species and nine other insect species, respectively. The analysis suggested TIMEOUT and TIMELESS proteins fall out into their respective clades (Figure 2). Pv_TIMEOUT was located in the same clade as TIMEOUT proteins from other Pteromalidae parasitoids, and the other TIMEOUTs, deriving from four families of parasitoid wasps, were separated into four family-specific clusters (Figure 2).

### 3.7. CRY2

**Figure 3:**
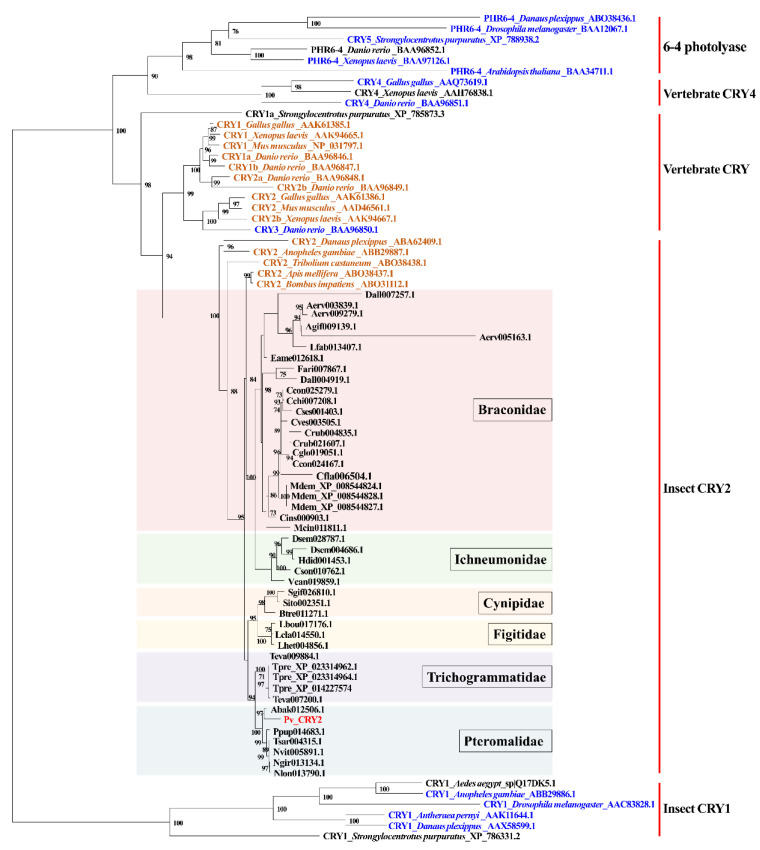
Phylogenetic relationships of DNA photolyases and CRYs. The phylogenetic tree of amino acid sequences was constructed using the maximum likelihood method. The best model is LG+I+G4. Bootstrap values below 70% were removed from the phylogenetic tree. The functional character is mapped onto this phylogenetic tree based on [1]. Orange letters indicate the proteins that can repress CLK: CYC (BMAL)-mediated transcription in cell culture. Blue letters indicate the proteins that lack this ability in cell culture. Black and red letters indicate the proteins whose transcriptional repressive activity remain unknown. The tip labels of parasitoid wasps show the protein names in InsectBase 2.0. Species information for parasitoid wasps is listed in Table S1 and other species are selected based on [1], whose tip labels consist of protein names, species names and Genbank numbers.

Pv_CRY2 shared 86% identity (BlastP, E-value = 0) with the CRY1 isoform X3 identified from *N. vitripennis* (Table 1). However, this protein is listed as ‘CRY1′ in NCBI despite clearly being CRY2 [28]. Pv_CRY2 contains a DNA photolyase and a flavin adenine dinucleotide (FAD) binding domain (Figure S8). The CLK: BMAL interaction domains (RD-1, RD-2a, and RD-2b) present in mice are also found in *P. vindemmiae* [49], as well as a coiled-coil domain. A conserved NLS, which is necessary for CRY nuclear localization, is also found within the RD-2b domain [50] (Figure S8). For the phylogenetic analysis of insect CRY1 and CRY2 proteins, we chose 6-4 photolyases and vertebrate CRY4 since they are most closely related to insect CRY1 and CRY2 phylogenetically [1]. Consistent with the previous results [1], the phylogenetic tree of CRY/DNA photolyase proteins mapped with the functional character revealed that all vertebrate CRY and insect CRY2 proteins had repressive transcriptional activity except for CRY3 from *Danio rerio*. All CRY2 proteins from parasitoid wasps clustered within the insect CRY2 group, which have been reported to repress CLK: CYC transcription in cell culture [1]. Pv_CRY2 clusters with corresponding proteins from other Pteromalidae parasitoids and the other CRY2 proteins from five families of parasitoid wasps were separated into five clusters to be family-specific (Figure 3). The evolutionary tree showed that the repressive transcriptional ability of insect CRY2 evolved from a photolyase-like ancestral gene lacking the ability to function as a transcriptional suppressor, and Pv_CRY2 might function as a transcriptional suppressor of CLK: CYC-mediated transcription like other insect CRY2 proteins. 

### 3.8. VRILLE and PDP1

Both Pv_VRILLE and Pv_PDP1 are members of the basic leucine zipper (bZIP) transcription factors [18]. Pv_VRILLE and Pv_PDP1 shared 77.95% identity (BlastP, E-value = 1 × 10^−163^) with nuclear factor interleukin-3-regulated protein (a vertebrate homolog of VRILLE protein) from *Melipona quadrifasciata*, and 99% identity (BlastP, E-value = 0) with the hepatic leukemia factor (a vertebrate homolog of PDP1 protein) isoform X9 from *N. vitripennis* (Table 1). Both Pv_VRILLE and Pv_PDP1 contain bZIP domains, which are essential for their DNA-binding function in the *Clk* gene promoter to regulate *Clk* transcription [18,51] (Figures S9 and S10). The evolutionary trees show that both Pv_VRILLE and Pv_PDP1 clustered with corresponding proteins from other Pteromalidae parasitoids. The other VRILLE and PDP1 proteins from different families of parasitoid wasps were separated into their respective clusters to be family-specific, with the exception of a PDP1 protein (Lcla007363.1) from *Leptopilina clavipes* (Hymenoptera: Figitidae), which clustered into the Pteromalidae group (Figures S11 and S12). 

### 3.9. CWO

Pv_CWO displayed 64% identity (BlastP, E-value = 1 × 10^−153^) with the transcription factor cwo isoform X2 from *Colletes gigas* (Table 1). Pv_CWO contains two highly conserved regions that are characteristic of known CWO proteins, namely, the bHLH and Hairy Orange domains (Figure S13) [52]. The bHLH domain is responsible for binding to DNA [43]. The Hairy Orange is an important functional domain in the *Drosophila* proteins Hesr-1, Hairy, and Enhancer of Split, which play a key role in inhibiting specific transcriptional activators [53]. According to the evolutionary tree, all CWO proteins were grouped into six major clusters to be family-specific and Pv_CWO clusters with corresponding proteins from other Pteromalidae parasitoids (Figure S14).

### 3.10. Expression Profiles of Clock Genes in P. vindemmiae Females and Males

**Figure 4:**
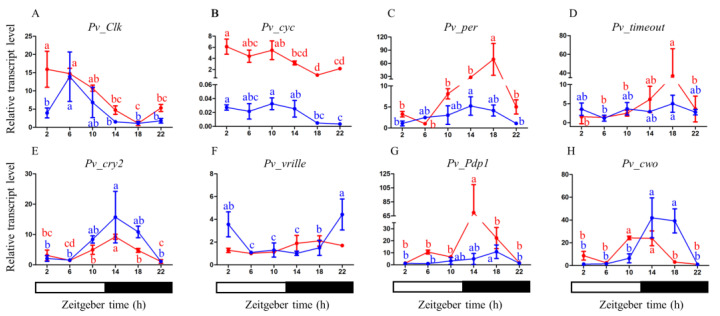
qPCR results showing the mRNA abundance levels of the eight clock genes in females (solid red lines) and males (solid blue lines) (**A**–**H**). Data are presented as the means ± standard deviation. The different letters are significantly different based on one-way analysis of variance (ANOVA) and Tukey’s test with differences considered significant at *p* < 0.05.

We obtained daily expression profiles of eight core clock genes in the heads of *P. vindemmiae* females and males. In the heads of female adults, *Pv_Clk* and *Pv_cyc* mRNA showed similar daily oscillations, with a peak in the early morning (ZT 2) and a trough at midnight (ZT 18) (Figure 4A,B). In contrast, *Pv_per*, *Pv_timeout*, *Pv_cry2*, *Pv_Pdp1*, and *Pv_cwo* mRNA levels were in an almost anti-phase to those of *Pv_clk* and *Pv_cyc*. *Pv_per* and *Pv_timeout* mRNA levels varied over time, with a peak at ZT 18 (Figure 4C,D). *Pv_vrille* showed a similar expression pattern to *Pv_per* and *Pv_timeout*, with no significant circadian changes detected (Figure 4F). *Pv_cry2* and *Pv_Pdp1* mRNA levels cycled in the heads of female adults with a peak at early night (ZT 14) and a trough at late night (ZT 22) and early morning (ZT 2), respectively (Figure 4E,G). *Pv_cwo* mRNA levels peaked between ZT 10 and 14 and declined to low levels between ZT 18 and 8 (Figure 4H).

The expression profiles of the same core clock genes were also measured In the heads of male adults. *Pv_Clk* and *Pv_cyc* mRNA levels were low during the night and reached their maxima at ZT 6, ZT 2, and ZT10 during the day, respectively (Figure 4A,B). *Pv_per*, *Pv_timeout*, *Pv_cry2*, *Pv_Pdp1*, and *Pv_cwo* mRNA levels were almost in anti-phase with those of *Pv_clk* and *Pv_cyc*. *Pv_per* and *Pv_cry2* transcript levels were low during the day, started to increase in the late day, and reached their maxima at ZT 14 (Figure 4C,E). *Pv_timeout* and *Pv_Pdp1* mRNA levels peaked at midnight (ZT 18) (Figure 4D,G). The peak levels of *Pv_vrille* mRNA from males occurred late at night or early morning (ZT 22-2) and decreased during the day (Figure 4F). *Pv_cwo* mRNA levels peaked between ZT 14 and 18 and declined to low levels between ZT 22 and 10 (Figure 4H). The qPCR results show differences in the clock gene expression patterns between female and male individuals. In males, the expression peaks of *Pv_Clk*, *Pv_Pdp1*, and *Pv_cwo* were delayed for several hours compared to females. *Pv_vrille* showed no significant rhythmic expression in females, while it peaked at late night and early morning in males.

## 4. Discussion

In parasitoid wasps, different circadian activities are performed at specific times to maximize fitness gains. *P. vindemmiae* oviposition had three peaks in the early morning, late day or early night, and late night (Figure 1C), while mating peaked at midday with no overlap with the oviposition activity (Figure 1B). In insects, repeated mating attempts by males can result in harassment of females and cause physical injuries, reduced foraging efficiency, and energy expenditure. Harassment by males can also interfere with the process of oviposition and reduce the longevity and fecundity of females [54]. No overlap between oviposition and mating peaks suggested that males were not able to disturb females during oviposition, indicating that *P. vindemmiae* has developed strategies to perform different life functions at specific times for maximum fitness gain. A similar situation has also been observed in *T. triozae* [9] and *E. warrae* [8].

Sex differences in circadian behavior have been observed in parasitoids. Firstly, a distinct difference between males and females in circadian activity is the time at which activity begins, which has been observed in *N. giraulti* [55], *Trichogramma* species [5,56], and *Encarsia formosa* [57]. Secondly, there is a difference in activity duration and intensity between males and females. For example, *T. brassicae* males have been observed to be less active than females since their phase of activity is shorter and their hourly activities fewer [58]. The daily rhythm of emergence also varied according to sex. It has been reported that males emerge before females (protandry) in some parasitoids [5]. In this study, the results show that *P. vindemmiae* males started to emerge half an hour earlier than females at ZT 22 (Figure 1A). The advantage of parasitoid wasp protandry is that the males emerge first, so they have a greater opportunity to mate with females. For females, protandry can reduce the time between emergence and mating, thereby reducing the risk of death before reproduction.

Knowledge of the circadian activity of parasitoid wasps can contribute to decisions on field release. For diurnal parasitoids, it is better to release the parasitoid wasps in the morning [9,59]. The circadian oviposition patterns of *Diachasmimorpha longicaudata* and *Doryctobracon crawfordi* overlap. The control efficacy is reduced when both parasitoid wasps are simultaneously released as a result of substantial competition [60]. Our study revealed the circadian activity of *P. vindemmiae* and provides information for the enhancement of its biological control. For instance, we may achieve better results if we carry out field release of mated *P. vindemmiae* females instead of newly emerged females in the early morning when environmental conditions are more favorable to avoid affecting the sex ratio of offspring due to insufficient mating because most *P. vindemmiae* female oviposition occurs in the early morning earlier than the peak they mate at noon.

**Figure 5:**
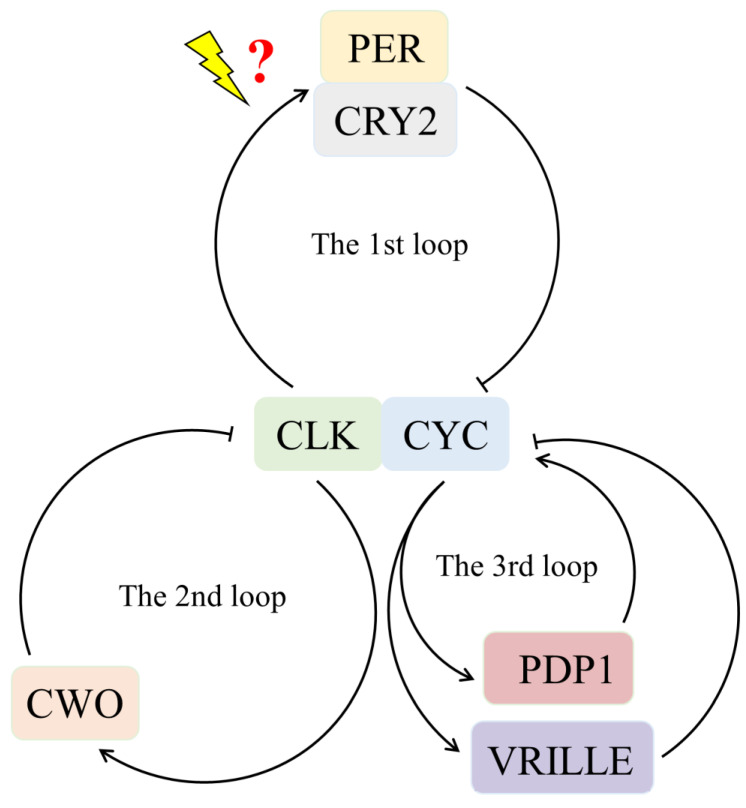
A hypothetical circadian clock model for *Pachycrepoideus vindemmiae*.

The absence of the *timeless* and *cry1* genes in parasitoid wasps suggests that their circadian clock system is distinct from that of other non-Hymenoptera insects such as *Drosophila*. While CRY1 is light-sensitive and does not exhibit transcriptional repressive activity, CRY2, a vertebrate-like protein, is a potent transcriptional repressor of CLK: CYC-mediated transcription but is not light-sensitive. These two *cry* genes give rise to different types of circadian clocks. Light-sensing in different types of circadian clocks is mediated by different mechanisms, leading to the synchronization of the clock via TIMELESS degradation [1]. However, Hymenoptera species, including parasitoid wasps, have lost both CRY1 and TIMELESS proteins, implying a completely novel light input mechanism for their circadian clocks [16,25,61]. Figure 5 presents a circadian clock model for *P. vindemmiae* that comprises three interlocked autoregulatory transcriptional-translational feedback loops. In the first major loop, heterodimers formed by the products of *Clk* and *cyc* genes activate the transcription of *per* and *cry2*. Subsequently, PER/CRY2 enter the nucleus to inhibit their own transcription by repressing CLK/CYC transcriptional activity. Because of the absence of CRY1, the clock uses a new light input pathway for entrainment. The second loop consists of the genes *Clk*, *cyc*, *vrille*, and *Pdp1*. CLK/CYC activates the transcription of *vrille* and *Pdp1* leading VRILLE to accumulate earlier than PDP1, which then suppresses *Clk* transcription. PDP1 accumulates later and triggers *Clk* transcription. Finally, CWO modulates the amplitude of the clock in the third loop.

Studies examining the expression patterns of core clock genes have demonstrated the diversity of the molecular clock machinery in insects. *Pv_Clk* mRNA in females exhibits a similar daily oscillation to *Clk* in *D. melanogaster*, peaking in the early morning [62] (Figure 4A), while *Clk* shows no significant rhythmic expression in *Solenopsis invicta* [19], *A. mellifera* [24] or *Gryllus bimaculatus* [18]. *Pv_cyc* mRNA levels are low during the night and reach their maxima in the early morning (Figure 4B), while *cyc* shows no significant rhythmic expression in *D. melanogaster* males [62] or *N. vitripennis* females [29]. In hymenopteran insects, *timeout* mRNA levels in both *P. vindemmiae* (Figure 4D) and *A. mellifera* [24] peak at midnight, while *timeout* mRNA levels in *S. invicta* are almost in anti-phase to these [19]. *Pv_cry2* mRNA cycles in the heads of female adults peak in the early night (Figure 4E), similar to those of *A. mellifera* females, while *cry2* mRNA levels peak at late night in *S. invicta* [19] and *N. vitripennis* [29]. However, *Pv_per* mRNA levels vary over time, with a peak at midnight (Figure 4C), similar to those of *D. melanogaster* [62], *S. invicta* [19], *A. mellifera* [24], and *G. bimaculatus* [18]. Overall, the expression profiles of core clock genes in *P. vindemmiae*, such as *Pv_Clk*, *Pv_cyc, Pv_per*, *Pv_timeout*, and *Pv_cry2*, are more similar to those of *A. mellifera* than other insects. To further investigate the diversity of circadian clock mechanisms, the expression patterns of clock genes need to be analyzed in more insect species, and the functions of the clock genes should be studied using molecular genetic techniques.

Although there have been only a few comparative studies on the expression patterns of female and male clock genes, previous research has shown that some clock genes are expressed consistently in both sexes [13,18,51], while others are quite variable [63,64]. Sex differences in circadian clock gene expression may arise from environmental variability and gene functions. In *Ceratosolen solmsi*, a pollinating wasp that has an obligate mutualism with fig trees, timeout is only rhythmically expressed in females that are dispersing from the fig syconium, while it is arrhythmic in males and females inside the syconium [63]. Among the core clock genes, *per* shows the largest divergence between the sexes in *C. solmsi*. Moreover, the mRNA levels of *per* are closely correlated with emergence rates at specific time intervals in both male and female wasps. The present study detected sexual dimorphism in the clock gene expression in *P. vindemmiae*, which suggests that these clock genes with different expression patterns in females and males may play a key role in protandry in *P. vindemmiae* (Figure 1A). More research on sexual dimorphism in clock gene expression is needed to reveal the extensive diversity of gene functions under various environmental conditions.

Although the molecular oscillatory mechanisms consisting of a set of clock genes have been extensively studied in some model insects [16,21,65,66], how the circadian clocks control daily rhythms in physiology and behavior via mechanisms that regulate gene expressions remains unknown. In *Anopheles* species, clock genes affect swarming and mating behavior by regulating the gene *desat1*, a dehydrogenase gene involved in the synthesis of a hydrocarbon on the body surface of male mosquitoes, which is a component of the sex pheromone of *Anopheles* [67]. Our present study characterized the circadian activity and putative clock genes in *P. vindemmiae*; however, the mechanisms by which the clock genes regulate circadian behaviors such as emergence, mating, and oviposition require further research.

## Figures and Tables

**Table 1:** Clock genes found in *Pachycrepoideus vindemmiae*.

Protein Name	Molecular Weight (kDa)	Accession Number	Blast Information (E-Value; Genbank No.; Species)
Pv_CLK	84.49	OQ145164	0.0; XP_032452987.1; *Nasonia vitripennis*
Pv_CYC	107.74	OQ145166	0.0; XP_008215805.1; *N. vitripennis*
Pv_PER	134.99	OQ145167	0.0; XP_008209246.1; *N. vitripennis*
Pv_TIMEOUT	69.32	OQ145168	0.0; XP_031783081.1; *N. vitripennis*
Pv_CRY2	65.57	OQ145169	0.0; XP_001606405.2; *N. vitripennis*
Pv_VRILLE	42.54	OQ145170	1 × 10^−163^; KOX68987.1; *Melipona quadrifasciata*
Pv_PDP1	27.71	OQ145171	0.0; XP_031779953.1; *N. vitripennis*
Pv_CWO	40.75	OQ145172	1 × 10^−153^; XP_043263793.1; *Colletes gigas*

**Table 2:** Clock genes identified from parasitoid wasps.

Species	Clock Gene									
	*Clk*	*cyc*	*per*	*tim*	*timeout*	*cry1*	*cry2*	*vrille*	*Pdp1*	*cwo*
*Apocrypta bakeri*	+	+	+	nd	+	nd	+	+	+	+
*Aphidius ervi*	+	+	+	nd	+	nd	+	+	+	+
*Aphidius gifuensis*	+	+	+	nd	+	nd	+	+	+	+
*Asobara japonica*	+	+	+	nd	+	nd	nd	+	+	+
*Belonocnema treatae*	+	+	+	nd	+	nd	+	+	+	+
*Cotesia chilonis*	+	+	+	nd	+	nd	+	+	+	+
*Cotesia congregata*	+	+	+	nd	+	nd	+	+	+	+
*Cotesia flavipes*	+	nd	+	nd	+	nd	+	+	+	+
*Copidosoma floridanum*	+	+	+	nd	+	nd	+	+	+	+
*Cotesia glomerata*	+	+	+	nd	+	nd	+	+	+	+
*Chelonus insularis*	+	+	+	nd	+	nd	+	+	+	+
*Cotesia rubecula*	+	nd	+	nd	+	nd	+	+	+	+
*Cotesia sesamiae*	+	nd	+	nd	+	nd	+	+	+	+
*Ceratosolen solmsi*	+	+	+	nd	+	nd	+	+	+	+
*Campoletis sonorensis*	+	+	+	nd	+	nd	+	+	+	+
*Cotesia vestalis*	+	+	+	nd	+	nd	+	+	+	+
*Diachasma alloeum*	+	+	nd	nd	+	nd	+	+	+	+
*Diadromus collaris*	+	nd	+	nd	+	nd	nd	+	+	+
*Diadegma semiclausum*	+	nd	+	nd	+	nd	+	+	+	+
*Eumacrocentrus americanus*	+	+	+	nd	+	nd	+	+	+	+
*Fopius arisanus*	+	+	+	nd	+	nd	+	+	+	+
*Gonatopus flavifemur*	+	nd	+	nd	+	nd	+	+	+	+
*Goniozus legneri*	+	+	+	nd	+	nd	+	+	+	+
*Hyposoter didymator*	+	+	+	nd	+	nd	+	+	+	+
*Leptopilina boulardi*	+	+	+	nd	+	nd	+	+	+	+
*Leptopilina clavipes*	+	+	+	nd	+	nd	+	+	+	+
*Lysiphlebus fabarum*	+	nd	+	nd	+	nd	+	+	+	+
*Leptopilina heterotoma*	+	+	+	nd	+	nd	+	+	+	+
*Macrocentrus cingulum*	+	+	+	nd	+	nd	+	+	+	+
*Microplitis demolitor*	+	+	+	nd	+	nd	+	+	+	+
*Nasonia giraulti*	+	+	+	nd	+	nd	+	+	+	+
*Nasonia longicornis*	+	+	+	nd	+	nd	+	+	+	+
*Nasonia vitripennis*	+	+	+	nd	+	nd	+	+	+	+
*Orussus abietinus*	+	+	+	nd	+	nd	+	+	+	+
*Pteromalus puparum*	+	+	+	nd	+	nd	+	+	+	+
*Pachycrepoideus vindemmiae*	+	+	+	nd	+	nd	+	+	+	+
*Sycophaga agraensis*	+	+	+	nd	+	nd	+	+	+	+
*Synergus gifuensis*	+	+	+	nd	+	nd	+	+	+	+
*Synergus itoensis*	+	+	+	nd	+	nd	+	+	+	+
*Trichogramma brassicae*	+	+	+	nd	+	nd	+	+	+	+
*Trichogramma evanescens*	+	+	+	nd	+	nd	+	+	+	+
*Trichogramma pretiosum*	+	nd	+	nd	+	nd	+	+	+	+
*Trichomalopsis sarcophagae*	+	+	+	nd	+	nd	+	nd	+	+
*Venturia canescens*	+	+	+	nd	+	nd	+	+	+	+

nd, not identified; +, identified.

## Data Availability

The data presented in this study are openly available in NCBI SRA database (https://www.ncbi.nlm.nih.gov/sra/PRJNA573955, accessed on 15 December 2022) and InsectBase 2.0 (http://v2.insect-genome.com/, accessed on 15 December 2022).

## Supplementary Materials

The following supporting information can be downloaded at: https://www.mdpi.com/article/10.3390/insects14050486/s1, Table S1: Circadian clock proteins in parasitoid wasps; Table S2: Primers used in qPCR analysis; Figure S1: Sequence alignment of CLK amino acid sequences; Figure S2: Phylogenetic relationships of CLKs from parasitoid wasps; Figure S3: Sequence alignment of alignment of of CYC amino acid sequences; Figure S4: Phylogenetic relationships of CYCs from parasitoid wasps; Figure S5: Sequence alignment of PER amino acid sequences; Figure S6: Phylogenetic relationships of PERs from parasitoid wasps; Figure S7: Sequence alignment of TIMEOUT amino acid sequences; Figure S8: (A) Structure and organization of Pv_CRY2; (B) Sequence alignment of CRY amino acid sequences; Figure S9: Sequence alignment of VRILLE amino acid sequences; Figure S10: Sequence alignment of PDP1 amino acid sequences; Figure S11: Phylogenetic relationships of VRILLE from parasitoid wasps; Figure S12: Phylogenetic relationships of PDP1 from parasitoid wasps; Figure S13: Sequence alignment of CWO amino acid sequences; Figure S14: Phylogenetic relationships of CWO proteins from parasitoid wasps. References [14,15,37,49,68,69] are cited in the supplementary materials.
