# Supplementary material for: Circadian Activity and Clock Genes in Pachycrepoideus vindemmiae: Implications for Field Applications and Circadian Clock Mechanisms of Parasitoid Wasps
Source: Insects. 2023 May 22;14(5):486. doi: 10.3390/insects14050486 (PMC10231066; doi:10.3390/insects14050486)
Supplement: Supplementary file 1 [file insects-14-00486-s001.zip › Supplementary figures.pdf]

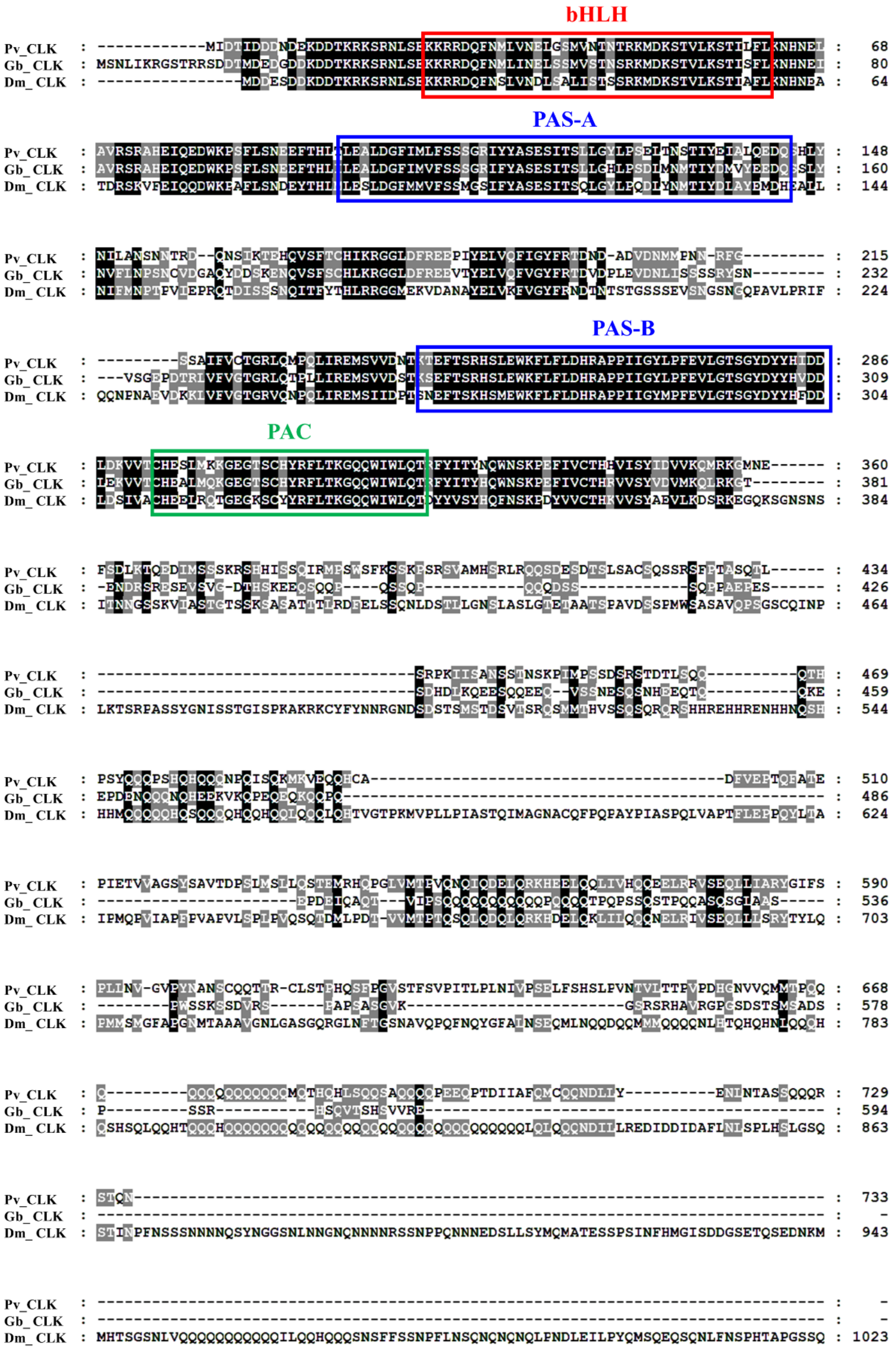

Figure S1. Sequence alignment of CLK amino acid sequences. bHLH (basic helix-loop-helix), PAS-A (Per-Arnt-Sim-A), PAS-B (Per-Arnt-Sim-B) and PAC (PAS associated C terminal) domains are shown based on the reference [1]. Pv_CLK: CLK from *Pachycrepoideus vindemmiae* (OQ145164), Gb_CLK: CLK from *Gryllus bimaculatus* (GenBank: AB738083), Dm_CLK: CLK from *Drosophila melanogaster* (GenBank: AF067207).

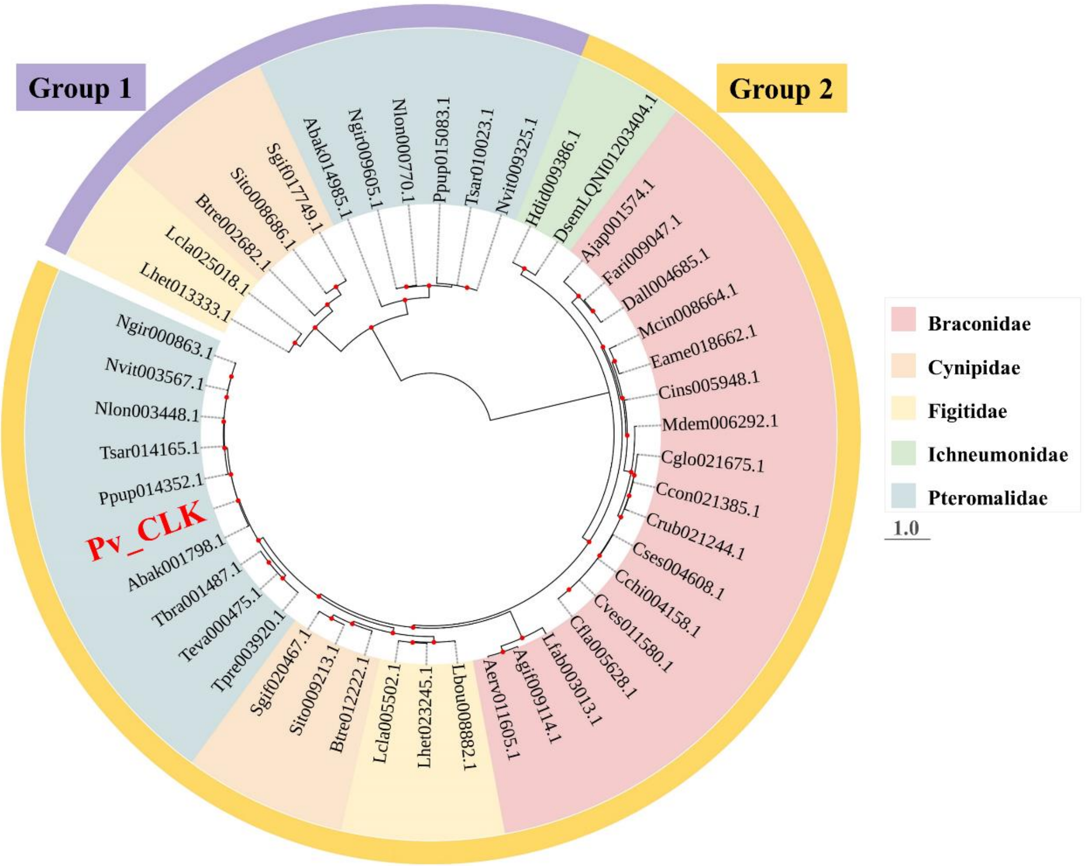

Figure S2. Phylogenetic relationships of CLKs from parasitoid wasps. The phylogenetic tree of amino acid sequences is constructed using the maximum likelihood method. The best model is JTT+G4. Red dots at the nodes denote bootstrap values greater than 500 from 1000 trials. Tip labels show the protein names in InsectBase 2.0. The species information is listed in Table S1.

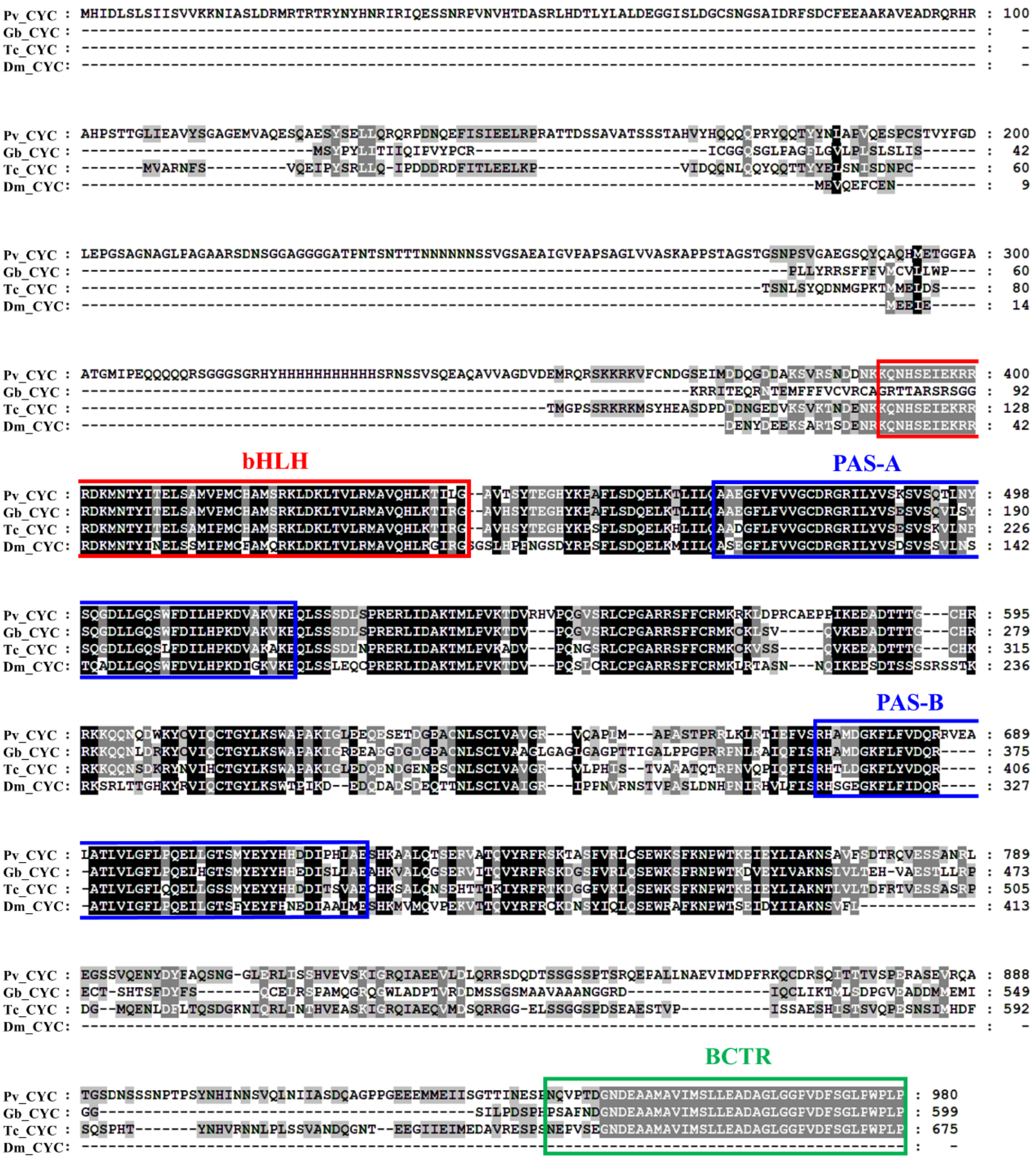

Figure S3. Sequence alignment of alignment of of CYC amino acid sequences. bHLH (basic helix-loop-helix), PAS-A (Per-arnt-Sim-A), PAS-B (Per-arnt-Sim-B) and brain and muscle aryl hydrocarbon receptor nuclear translocator- like C-terminal region (BCTR) domains based on the reference [2]. Pv_CYC: CYC from *Pachycrepoideus vindemmiae* (OQ145166), Gb_CYC: CYC from *Gryllus bimaculatus* (GenBank: BAN28450.1), Tc_CYC: CYC from *Tribolium castaneum* (GenBank: NP_001107795.1), Dm_CYC: CYC from *Drosophila melanogaster* (GenBank: AAF49107.1).

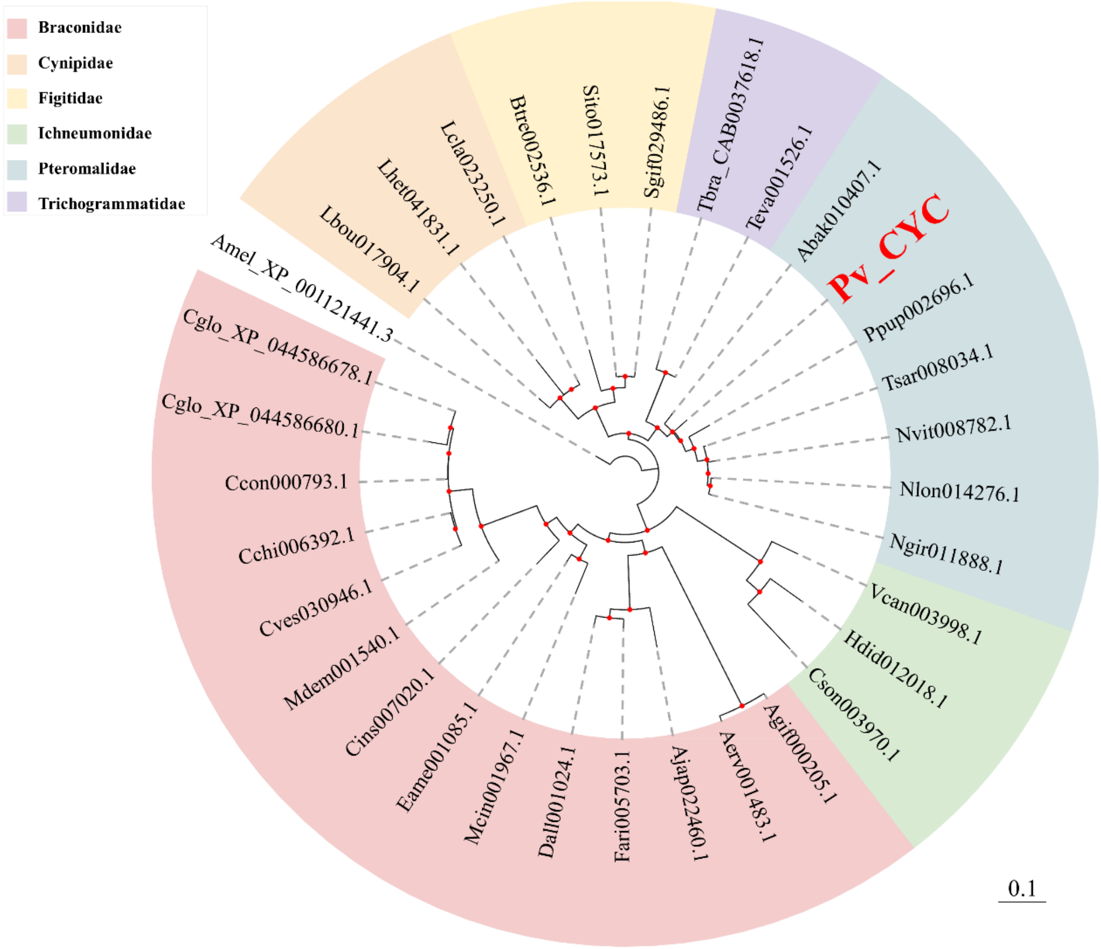

Figure S4. Phylogenetic relationships of CYCs from parasitoid wasps. The phylogenetic tree of amino acid sequences is constructed using the maximum likelihood method. The best model is JTT+G4. Red dots at the nodes denote bootstrap values greater than 500 from 1000 trials. This phylogenetic tree is rooted by ARNT-like protein 1 isoform X1 (Amel_XP_001121441.3) from *Apis mellifera*. Tip labels show the protein names in InsectBase 2.0. The species information is listed in Table S1.

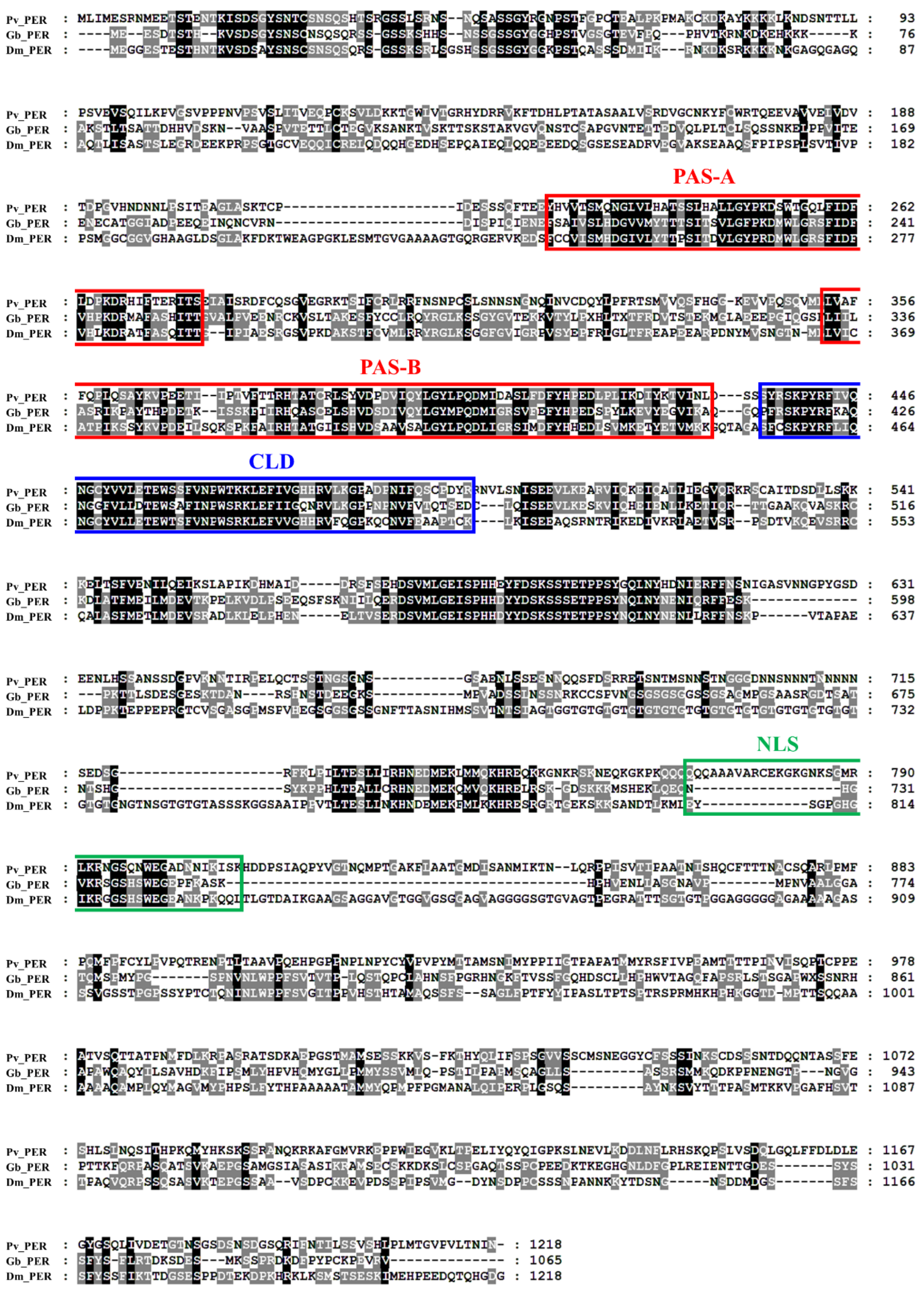

Figure S5. Sequence alignment of PER amino acid sequences. PAS-A (Per-arnt-Sim-A), PAS-B (Per-arnt-Sim-B), CLD (cytoplasmic localization domain) and NLS (nuclear localization signal) domains are shown based on the reference [3-4]. Pv_PER: PER from *Pachycrepoideus vindemmiae* (OQ145167), Gb_PER: PER from *Gryllus bimaculatus* (GenBank: BAG48878.1), Dm_PER: PER from *Drosophila melanogaster* (GenBank: AAF45804.1).

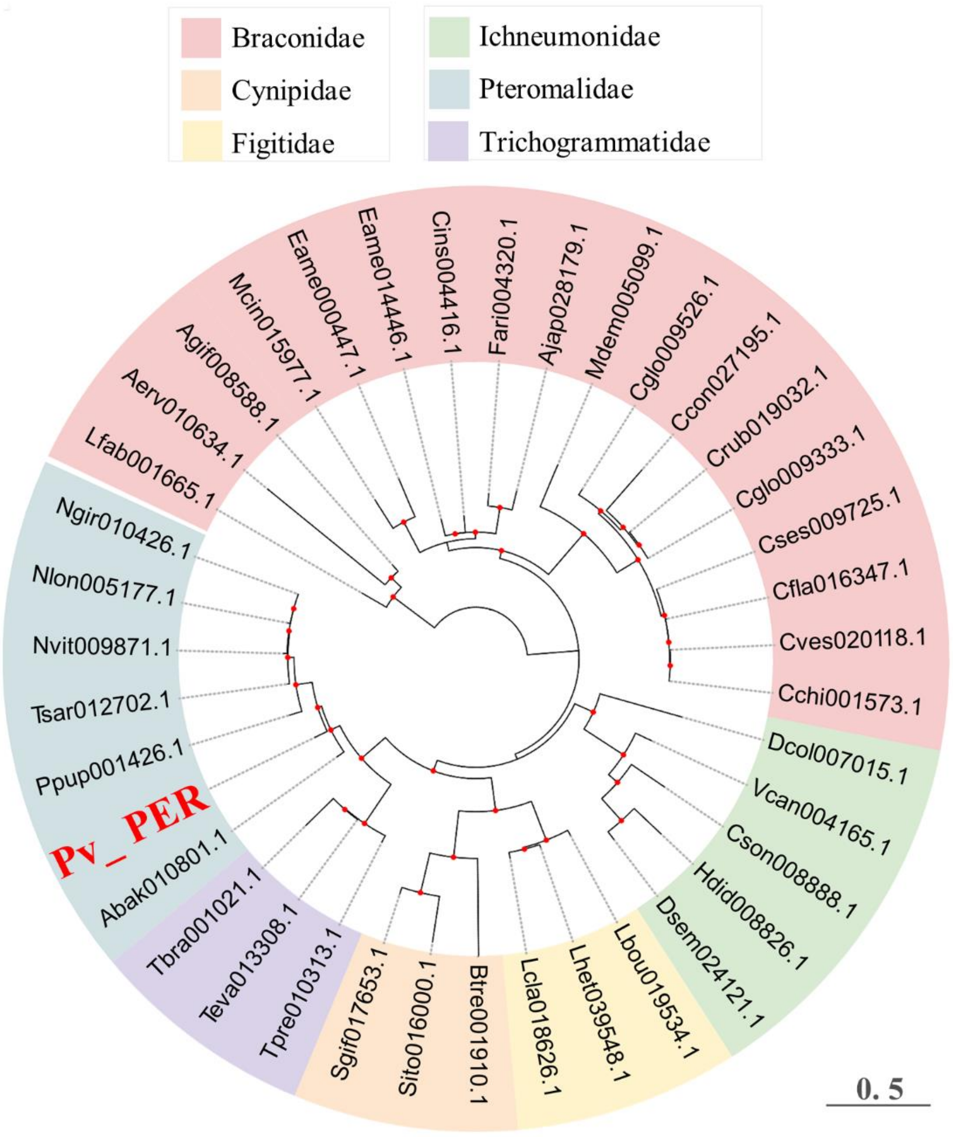

Figure S6. Phylogenetic relationships of PERs from parasitoid wasps. The phylogenetic tree is constructed using the maximum likelihood method. The best model is JTT+G4. Red dots at the nodes denote bootstrap values greater than 500 from 1000 trials. Tip labels show the protein names in InsectBase 2.0. The species information is listed in Table S1.

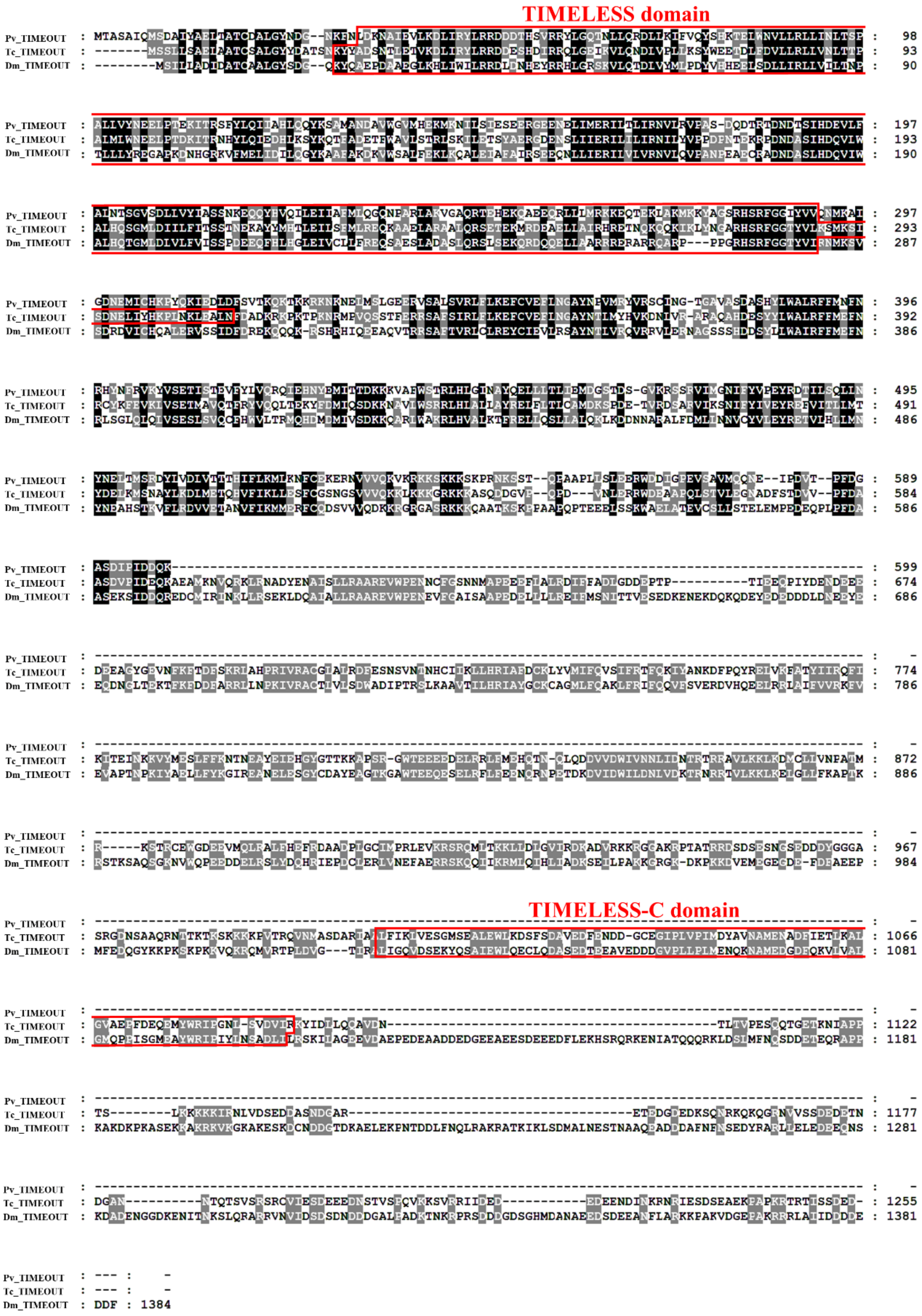

Figure S7. Sequence alignment of TIMEOUT amino acid sequences. TIMELESS and TIMELESS-C domains are shown according to CDD search [5]. Pv_TIMEOUT: TIMEOUT from *Pachycrepoideus vindemmiae* (OQ145168), Dm_TIMEOUT: TIMEOUT from *Drosophila melanogaster* (GenBank: AAF73481.1), Tc_TIMEOUT: TIMEOUT from *Tribolium castaneum* (GenBank: EEZ99220.1).

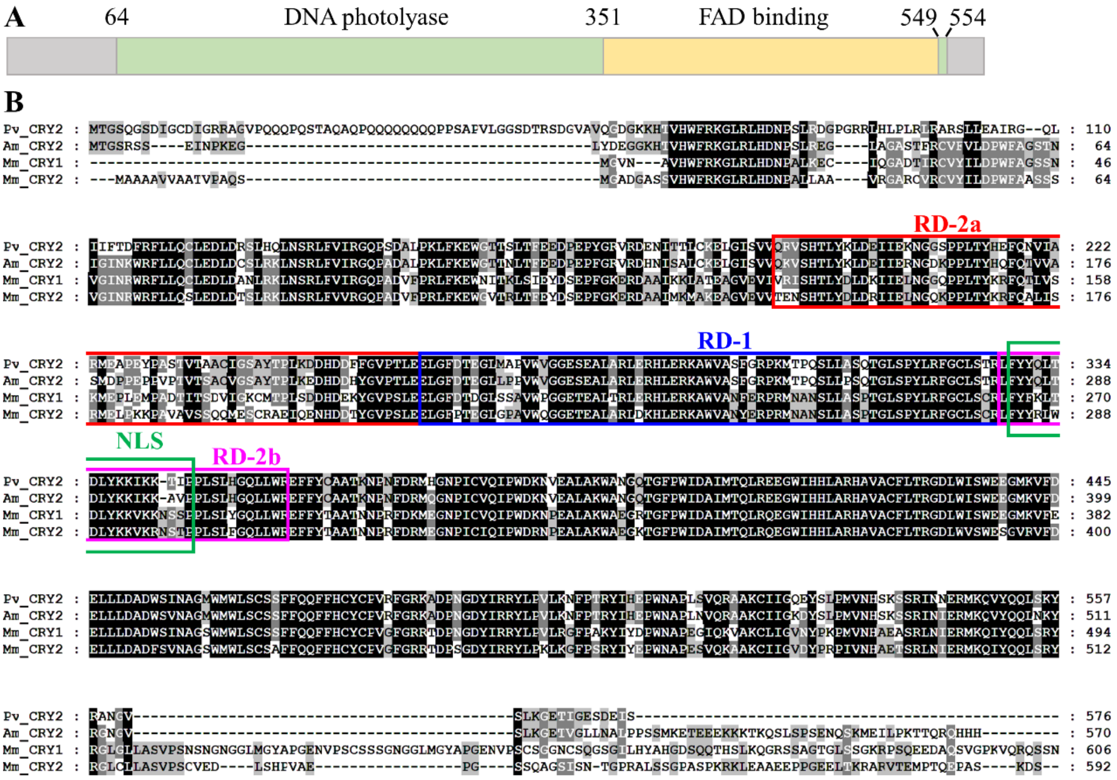

Figure S8. (**A**) Structure and organization of Pv_CRY2. DNA photolyase and flavin adenine dinucleotide (FAD) domains are indicated by green and yellow rectangles, respectively, according to CDD search [5]. (**B**) Sequence alignment of CRY amino acid sequences. RD-1, RD-2a, RD-2b, NLS (nuclear localization signal) and coiled-coil domains are shown based on the reference [6]. Pv_CRY2: CRY2 from *Pachycrepoideus vindemmiae* (OQ145169), Am_CRY2: CRY2 from *Apis mellifera* (GenBank: NP_001077099.1), Mm_CRY1: CRY1 from *Mus musculus* (GenBank: P97784.1), Mm_CRY2: CRY2 from *M. musculus* (GenBank: Q9R194.1).

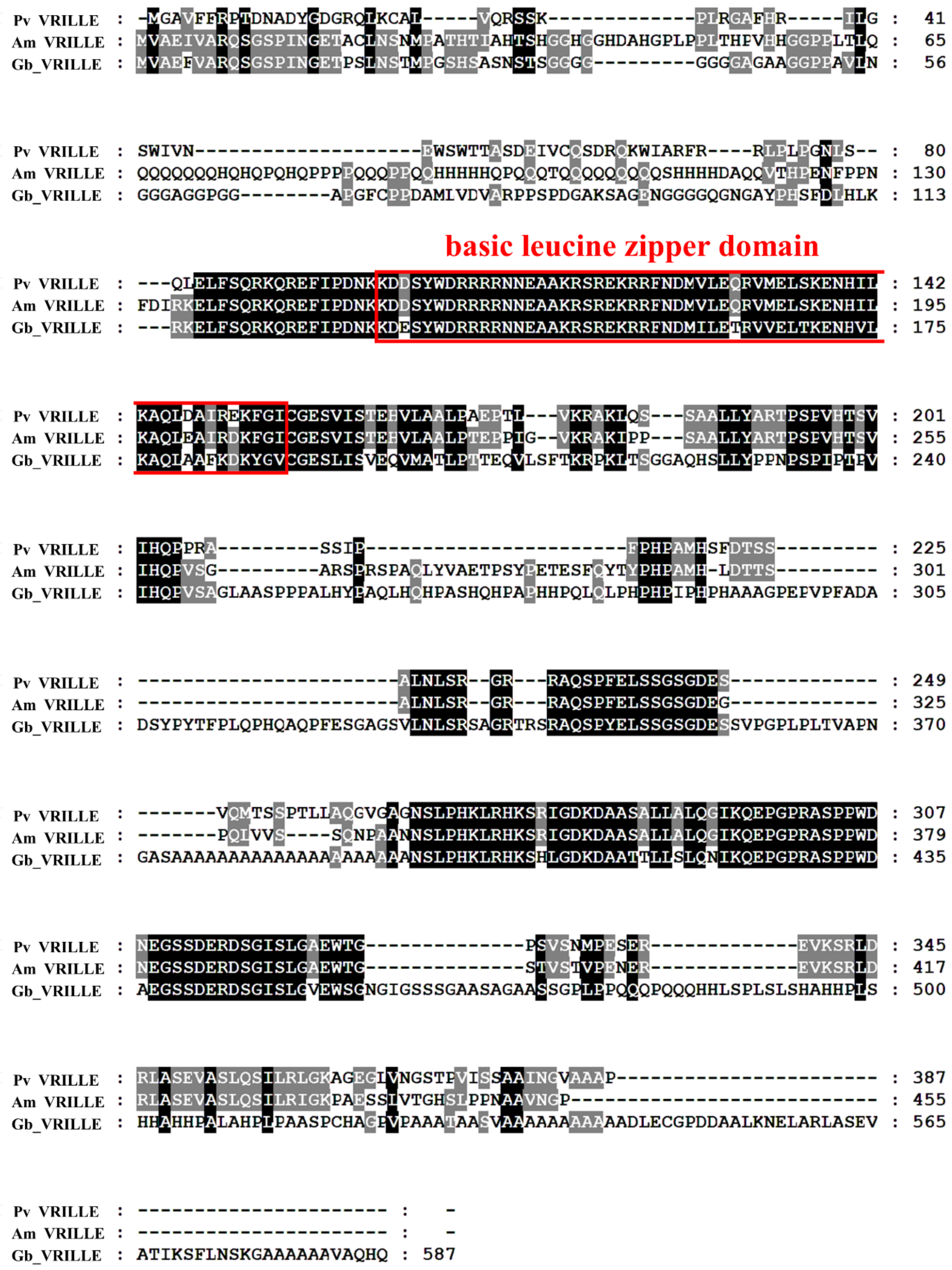

Figure S9. Sequence alignment of VRILLE amino acid sequences. bZIP (basic leucine zipper) domain is identified by CDD [5]. Pv_VRILLE: VRILLE from *Pachycrepoideus vindemmiae* (OQ145170), Am_VRILLE: VRILLE from *Apis mellifera* (GenBank: XP_393735.3), Gb_VRILLE: VRILLE from *Gryllus bimaculatus* (GenBank: BBQ05060.1).

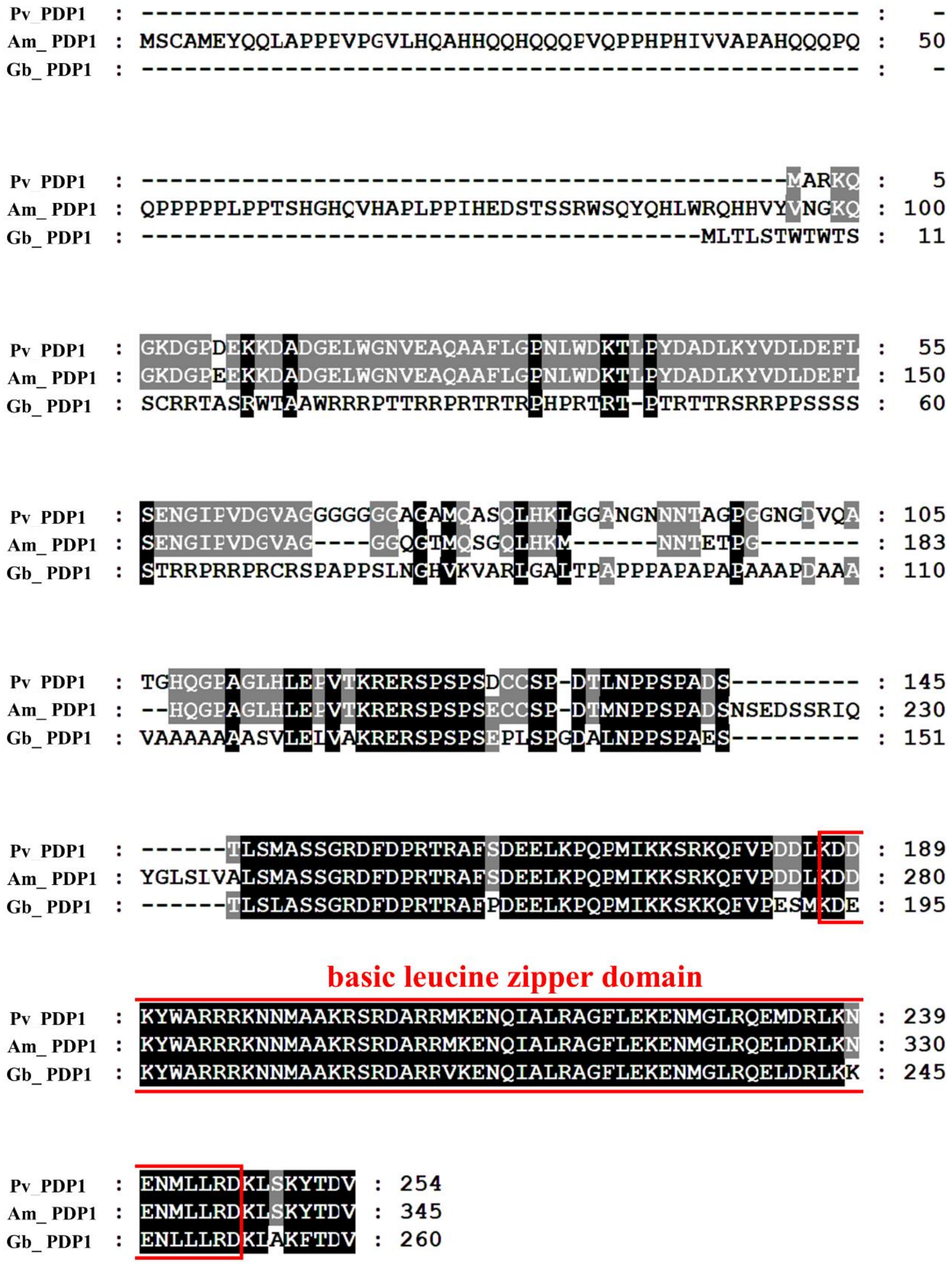

Figure S10. Sequence alignment of PDP1 amino acid sequences. bZIP (basic leucine zipper) domain is identified by CDD [5]. Pv_PDP1: PDP1 from *Pachycrepoideus vindemmiae* (OQ145171), Am_PDP1: PDP1 from *A. mellifera* (GenBank: XP_391997.3), Gb_PDP1: PDP1 from *G. bimaculatus* (GenBank: BBQ05061.2).

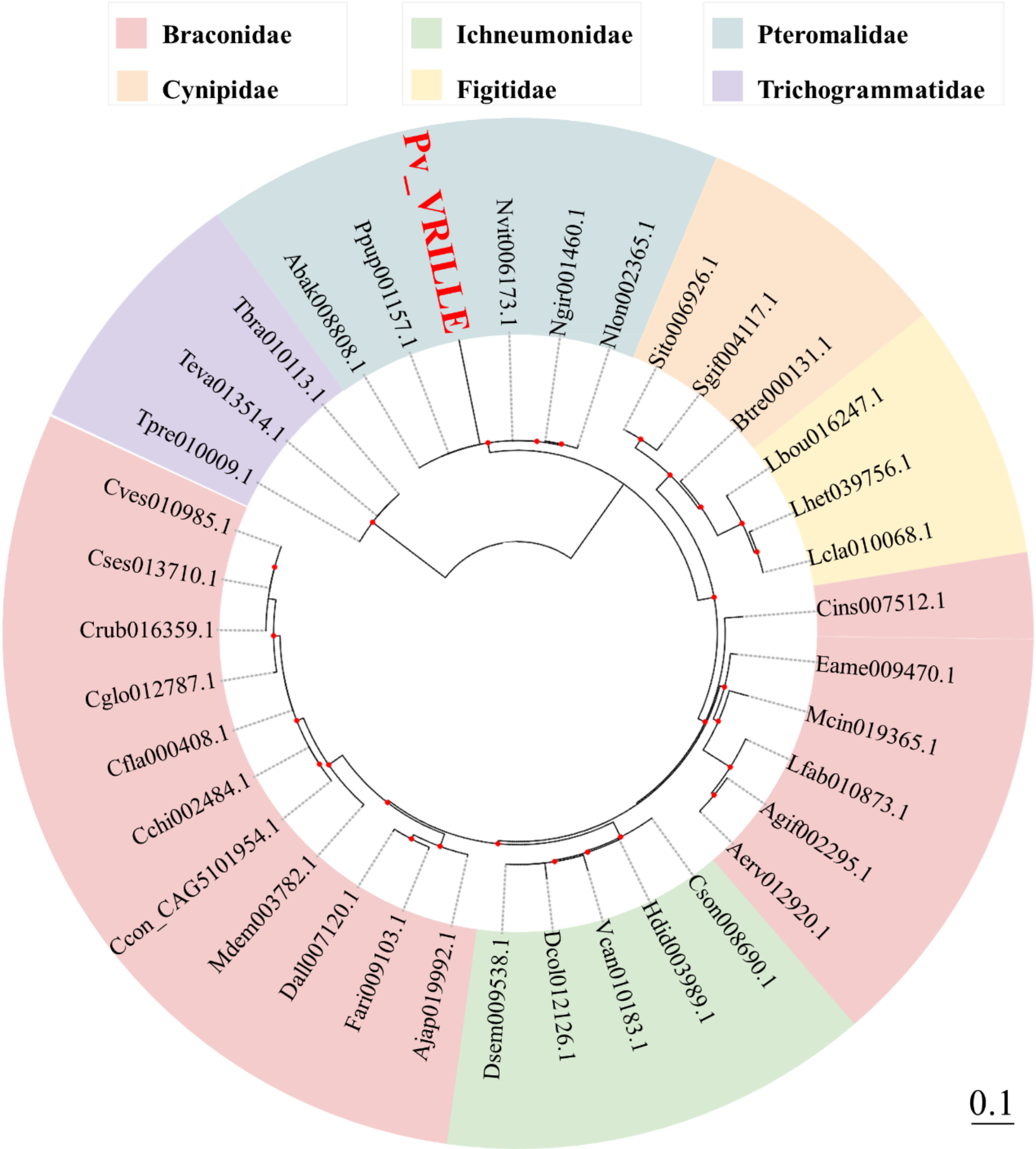

Figure S11. Phylogenetic relationships of VRILLE from parasitoid wasps. The phylogenetic tree is constructed using the maximum likelihood method. The best model is JTT+G4. Red dots at the nodes denote bootstrap values greater than 500 from 1000 trials. Tip labels show the protein names in InsectBase 2.0. The species information is listed in Table S1.

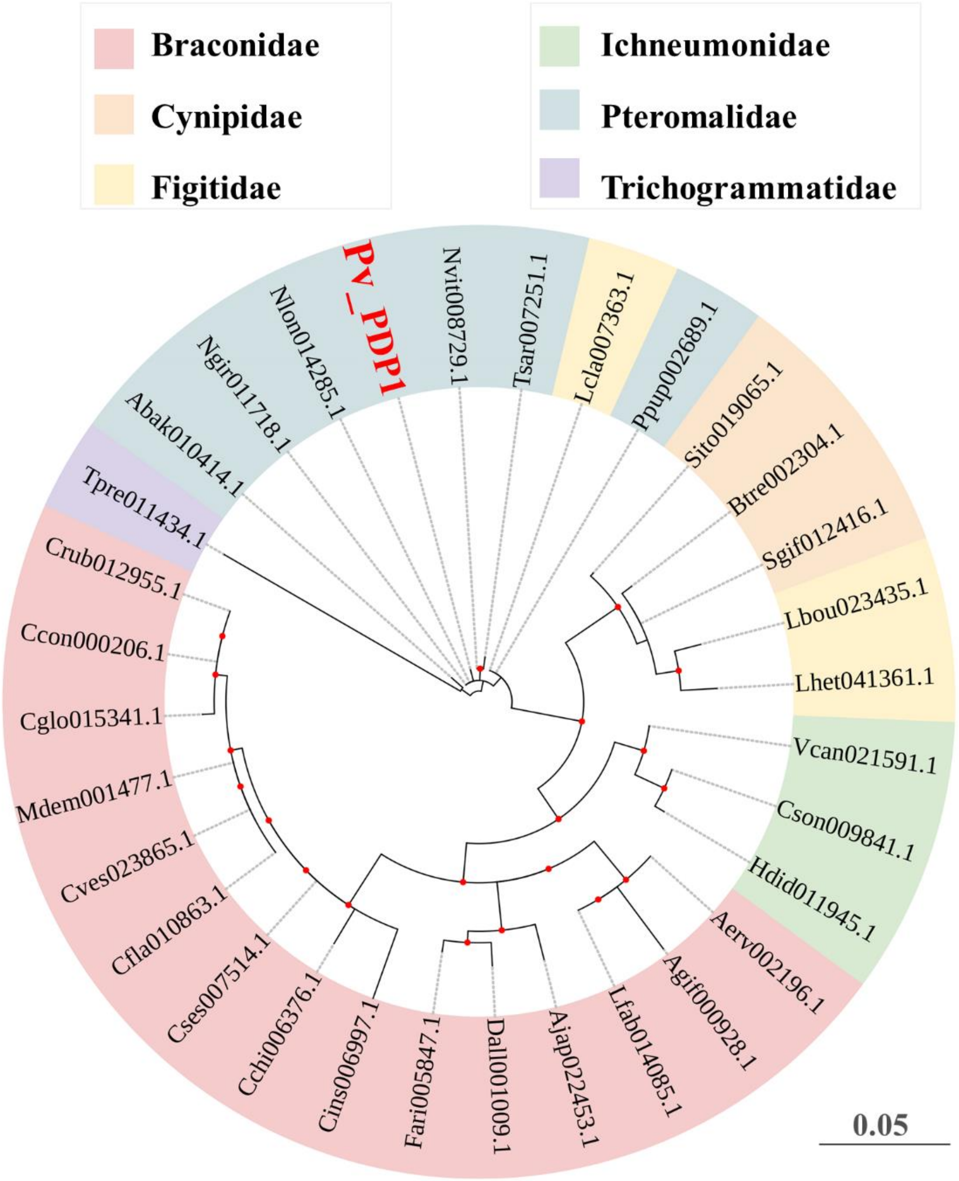

Figure S12. Phylogenetic relationships of PDP1 from parasitoid wasps. The phylogenetic tree is constructed using the maximum likelihood method. The best model is JTT+I+G4. Red dots at the nodes denote bootstrap values greater than 500 from 1000 trials. Tip labels show the protein names in InsectBase 2.0. The species information is listed in Table S1.

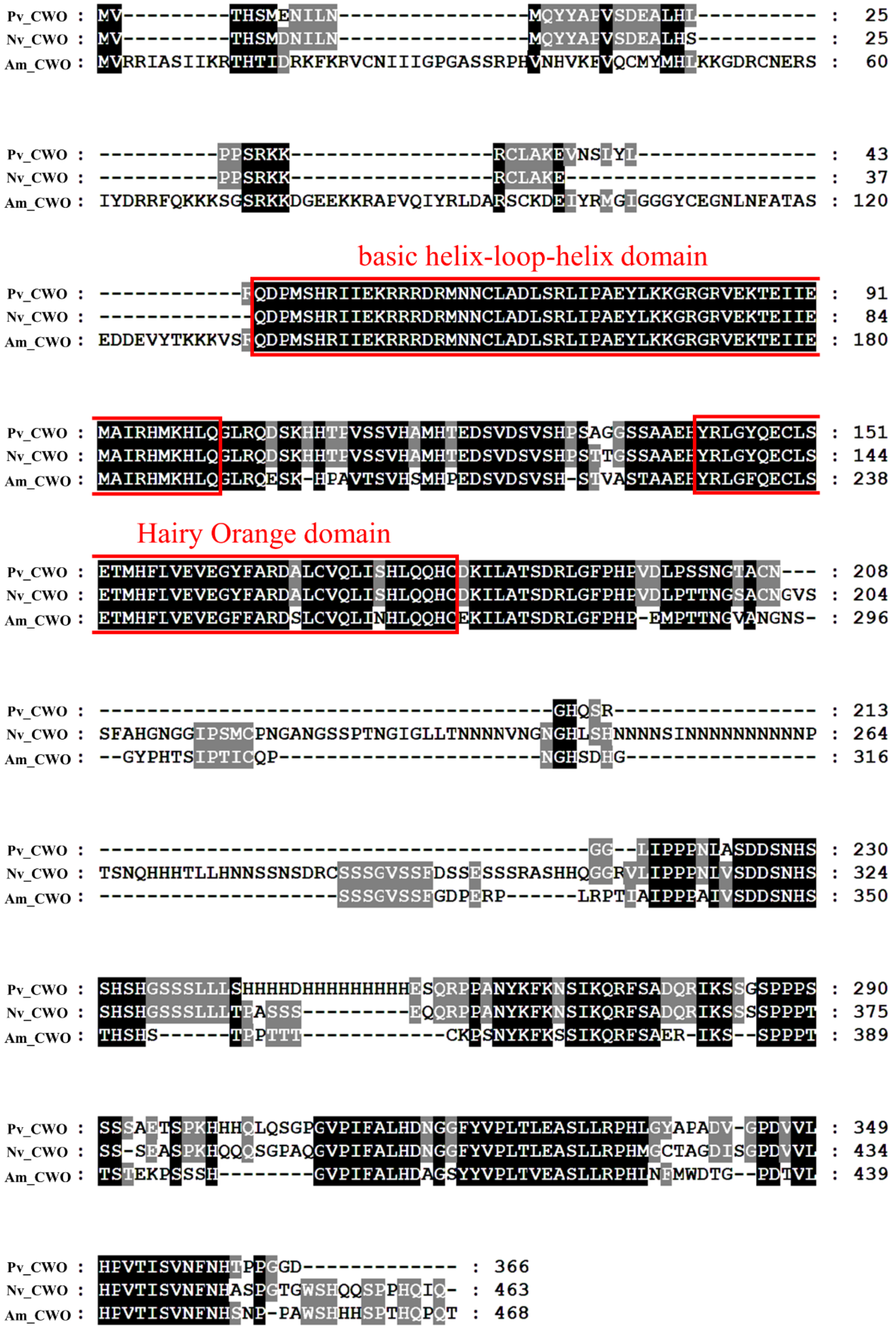

Figure S13. Sequence alignment of CWO amino acid sequences. basic helix-loop-helix (bHLH) and Hairy Orange domains are identified by CDD [5]. Pv_CWO: CWO from *Pachycrepoideus vindemmiae* (OQ145172), Nv_CWO: CWO from *Nasonia vitripennis* (GenBank: XP_003426877.1), Am_CWO: CWO from *Apis mellifera* (GenBank: GB52039).

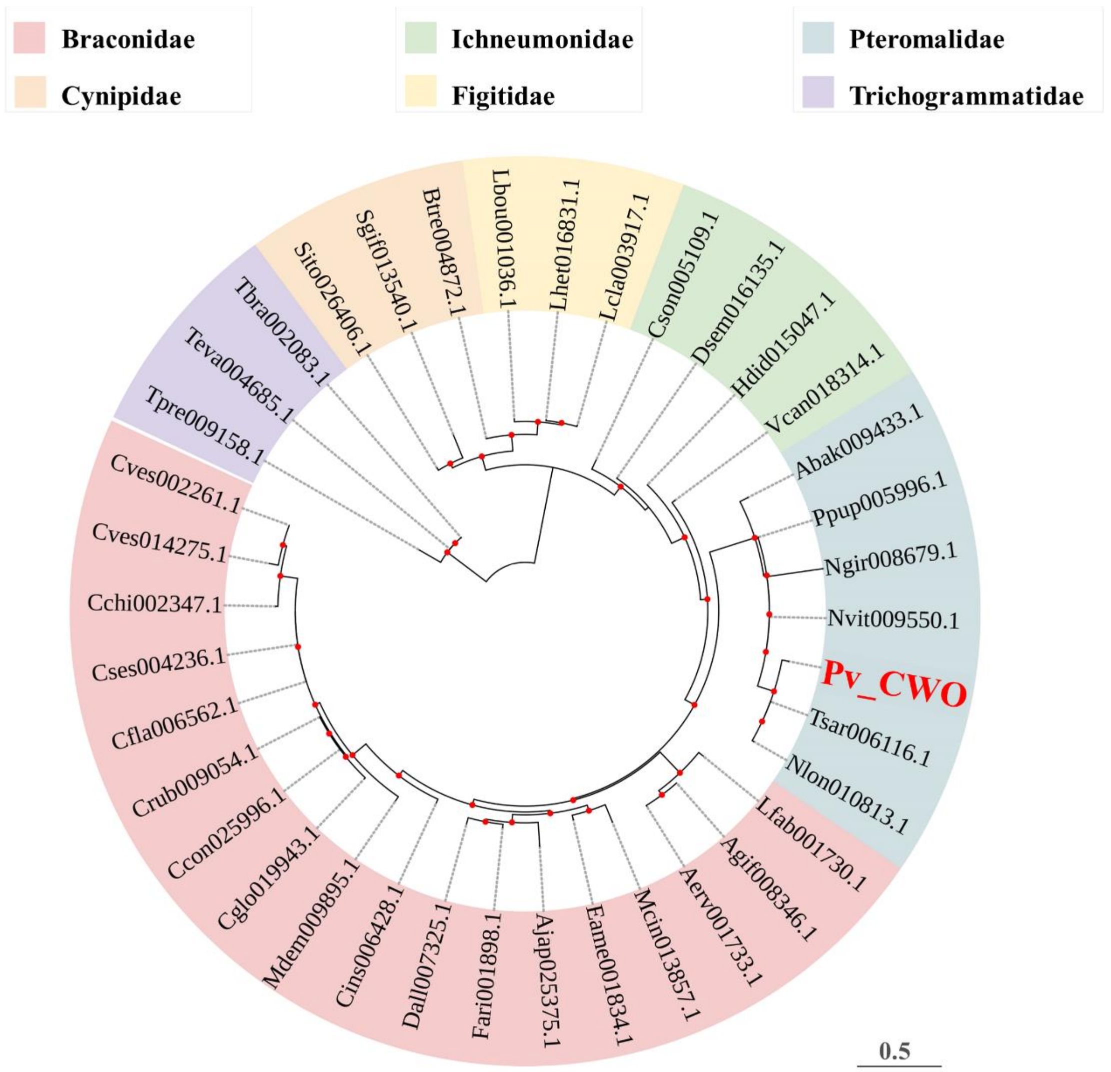

Figure S14. Phylogenetic relationships of CWO proteins from parasitoid wasps. The phylogenetic tree is constructed using the maximum likelihood method. The best model is JTT+G4. Red dots at the nodes denote bootstrap values greater than 500 from 1000 trials. Tip labels show the protein names in InsectBase 2.0. The species information is listed in Table S1.

References

1. Takekata, H.; Numata, H.; Shiga, S.; Goto, S.G. Silencing the circadian clock gene *Clock* using RNAi reveals dissociation of the circatidal clock from the circadian clock in the mangrove cricket. *J. Insect Physiol.* **2014,** *68*, 16-22.
2. Uryu, O.; Karpova, S.G.; Tomioka, K. The clock gene cycle plays an important role in the circadian clock of the cricket *Gryllus bimaculatus*. *J. Insect Physiol.* **2013,** *59*, 697-704.
3. Fergus, D.J.; Shaw, K.L. Circadian rhythms and *period* expression in the Hawaiian cricket genus *Laupala*. *Behav. Genet.* **2013,** *43*, 241-253.
4. Moriyama, Y.; Sakamoto, T.; Karpova, S.G.; Matsumoto, A.; Noji, S.; Tomioka, K. RNA interference of the clock gene *period* disrupts circadian rhythms in the cricket *Gryllus bimaculatus*. *J. Biol. Rhythm* **2008,** *23*, 308-318.
5. Marchler-Bauer, A.; Bo, Y.; Han, L.; He, J.; Lanczycki, C.J.; Lu, S.; Chitsaz, F.; Derbyshire, M.K.; Geer, R.C.; Gonzales, N.R.; Gwadz, M.; Hurwitz, D.I.; Lu, F.; Marchler, G.H.; Song, J.S.; Thanki, N.; Wang, Z.; Yamashita, R.A.; Zhang, D.; Zheng, C.; Geer, L.Y.; Bryant, S.H. CDD/SPARCLE:

functional classification of proteins via subfamily domain architectures. *Nucleic Acids Res.* **2017,** *45*, D200-D203.

6. Hirayama, J.; Nakamura, H.; Ishikawa, T.; Kobayashi, Y.; Todo, T. Functional and structural analyses of cryptochrome- Vertebrate cry regions responsible for interaction with the CLOCK: BMAL1 heterodimer and its nuclear localization. *J. Biol. Chem.* **2003,** *278*, 35620-35628.
