# Supplementary material for: Circadian Activity and Clock Genes in Pachycrepoideus vindemmiae: Implications for Field Applications and Circadian Clock Mechanisms of Parasitoid Wasps
Source: Insects. 2023 May 22;14(5):486. doi: 10.3390/insects14050486 (PMC10231066; doi:10.3390/insects14050486)
Supplement: Supplementary file 1 [file insects-14-00486-s001.zip › Table S1.pdf]

Table S1. Circadian clock proteins in parasitoid wasps

Species	CLK	CYC	PER	TIMEOUT	CRY2	VRILLE	PDP1	CWO
Apocrypta bakeri	Abak001798.1<br>Abak014985.1	Abak010407.1	Abak010801.1	Abak003066.1<br>Abak003069.1	Abak012506.1	Abak008808.1	Abak010414.1<br>Abak014499.1	Abak009433.1
Aphidius ervi	Aerv011605.1	Aerv001483.1	Aerv010634.1	Aerv002058.1<br>Aerv002093.1	Aerv003839.1<br>Aerv005163.1<br>Aerv009279.1	Aerv012920.1	Aerv002196.1<br>Aerv016047.1	Aerv001733.1
Aphidius gifuensis	Agif009114.1	Agif000205.1	Agif008588.1	Agif002420.1	Agif009139.1	Agif002295.1	Agif000928.1	Agif008346.1
Asobara japonica	Ajap001574.1	Ajap022460.1	Ajap028179.1	Ajap023887.1		Ajap019992.1	Ajap003846.1<br>Ajap022453.1	Ajap025375.1
Belonocnema treatae	Btre002682.1<br>Btre012222.1	Btre002536.1	Btre001910.1	Btre012312.1	Btre011271.1	Btre000131.1	Btre001161.1<br>Btre002304.1	Btre004872.1
Cotesia chilonis	Cchi004158.1	Cchi006392.1	Cchi001573.1	Cchi003716.1<br>Cchi003717.1	Cchi007208.1	Cchi002484.1	Cchi006376.1	Cchi002347.1
Cotesia congregata	Ccon021385.1	Ccon000793.1	Ccon027195.1	Ccon018683.1	Ccon025279.1<br>Ccon024167.1	Ccon_CAG5101954<br>.1	Ccon000206.1	Ccon025996.1
Cotesia flavipes	Cfla005628.1		Cfla016347.1	Cfla010270.1<br>Cfla013825.1	Cfla006504.1	Cfla000408.1	Cfla010863.1	Cfla006562.1
Copidosoma floridanum	Cflr008858.1	Cflr008974.1	Cflr003358.1	Cflr003252.1	Cflr004247.1	Cflr002281.1	Cflr010250.1	Cflr000081.1
Cotesia glomerata	Cglo021675.1	Cglo_XP_044586678.1<br>Cglo_XP_044586680.1	Cglo009333.1<br>Cglo009526.1	Cglo004163.1<br>Cglo020585.1	Cglo019051.1	Cglo012787.1	Cglo015341.1	Cglo019943.1
Chelonus insularis	Cins005948.1	Cins007020.1	Cins004416.1	Cins001721.1	Cins000903.1	Cins007512.1	Cins001808.1<br>Cins006997.1<br>Cins009491.1	Cins006428.1

Cotesia rubecula	Crub021244.1		Crub019032.1	Crub005177.1<br>Crub007184.1	Crub004835.1<br>Crub021607.1	Crub016359.1	Crub012955.1<br>Crub020266.1	Crub009054.1
Cotesia sesamiae	Cses004608.1		Cses009725.1	Cses015321.1<br>Cses016092.1	Cses001403.1	Cses013710.1	Cses007514.1	Cses004236.1
Ceratosolen solmsi	Csol001972.1	Csol006905.1	Csol006961.1	Csol000087.1	Csol004819.1	Csol003821.1	Csol001731.1<br>Csol006976.1	Csol008126.1
Campoletis sonorensis	Cson007249.1	Cson003970.1	Cson008888.1	Cson018992.1<br>Cson018994.1	Cson010762.1	Cson008690.1	Cson009841.1<br>Cson010604.1	Cson005109.1
Cotesia vestalis	Cves011580.1	Cves030946.1	Cves020118.1	Cves013030.1<br>Cves031101.1	Cves003505.1	Cves010985.1	Cves023865.1	Cves002261.1<br>Cves014275.1
Diachasma alloeum	Dall004685.1	Dall001024.1		Dall000860.1	Dall004919.1<br>Dall007257.1	Dall007120.1	Dall001009.1<br>Dall001905.1	Dall007325.1
Diadromus collaris	Dcol001446.1<br>Dcol041669.1		Dcol007015.1<br>Dcol019807.1	Dcol031634.1<br>Dcol034640.1		Dcol012126.1	Dcol008885.1<br>Dcol023729.1	Dcol027844.1
Diadegma semiclausum	DsemLQNI01173890<br>.1<br>DsemLQNI01203404<br>.1		Dsem024121.1	Dsem001288.1	Dsem004686.1<br>Dsem028787.1	Dsem009538.1	Dsem030959.1	Dsem016135.1
Eumacrocentrus americanus	Eame018662.1	Eame001085.1	Eame000447.1<br>Eame014446.1	Eame002359.1<br>Eame014621.1	Eame012618.1	Eame009470.1	Eame004163.1	Eame001834.1
Fopius arisanus	Fari009047.1	Fari005703.1	Fari004320.1	Fari004363.1	Fari007867.1	Fari009103.1	Fari005634.1<br>Fari005847.1	Fari001898.1
Gonatopus flavifemur	Gfla000945.1		Gfla009668.1	Gfla007985.1<br>Gfla008116.1	Gfla009811.1	Gfla003212.1	Gfla017971.1	Gfla020122.1
Goniozus legneri	Gleg001201.1	Gleg008040.1	Gleg006125.1	Gleg007998.1	Gleg006534.1	Gleg001577.1	Gleg000751.1	Gleg004982.1

				Gleg011592.1				
Hyposoter didymator	Hdid009386.1	Hdid012018	Hdid008826.1	Hdid000325.1	Hdid001453.1	Hdid003989.1	Hdid011945.1<br>Hdid013746.1	Hdid015047.1
Leptopilina boulardi	Lbou008882.1<br>Lbou023138.1	Lbou017904.1	Lbou019534.1	Lbou009639.1<br>Lbou010183.1<br>Lbou020194.1	Lbou017176.1	Lbou016247.1	Lbou012757.1<br>Lbou023435.1	Lbou001036.1
Leptopilina clavipes	Lcla005502.1<br>Lcla025018.1	Lcla023250.1	Lcla018626.1	Lcla009997.1<br>Lcla023072.1	Lcla014550.1	Lcla010068.1	Lcla007363.1<br>Lcla016153.1	Lcla003917.1
Lysiphlebus fabarum	Lfab003013.1		Lfab001665.1	Lfab011224.1<br>Lfab011248.1	Lfab013407.1	Lfab010873.1	Lfab013773.1<br>Lfab014085.1	Lfab001730.1
Leptopilina heterotoma	Lhet013333.1<br>Lhet023245.1	Lhet041831.1	Lhet039548.1	Lhet017194.1	Lhet004856.1	Lhet039756.1	Lhet024704.1<br>Lhet041361.1	Lhet016831.1
Macrocentrus cingulum	Mcin008664.1	Mcin001967.1	Mcin015977.1	Mcin007098.1	Mcin011811.1	Mcin019365.1	Mcin001107.1<br>Mcin001976.1<br>Mcin011070.1	Mcin013857.1
Microplitis demolitor	Mdem006292.1	Mdem001540.1	Mdem005099.1	Mdem007077.1	Mdemo_XP_008544824.1<br>Mdemo_XP_008544827.1<br>Mdemo_XP_008544828.1	Mdem003782.1	Mdem001477.1	Mdem009895.1
Nasonia giraulti	Ngir000863.1<br>Ngir009605.1	Ngir011888.1	Ngir010426.1	Ngir002622.1	Ngir013134.1	Ngir001460.1	Ngir009862.1<br>Ngir011718.1	Ngir008679.1
Nasonia longicornis	Nlon000770.1<br>Nlon003448.1	Nlon014276.1	Nlon005177.1	Nlon011621.1<br>Nlon011623.1	Nlon013790.1	Nlon002365.1	Nlon000740.1<br>Nlon014285.1	Nlon010813.1
Nasonia vitripennis	Nvit003567.1<br>Nvit009325.1	Nvit008782.1	Nvit009871.1	Nvit001277.1	Nvit005891.1<br>Nvit_XP_016841836.1<br>Nvit_XP_001606405.2<br>Nvit_XP_016841837.1	Nvit006173.1	Nvit008270.1<br>Nvit008729.1	Nvit009550.1

					Nvit_XP_032456530.1<br>Nvit_XP_032456531.1<br>Nvit_XP_008204454.1			
Orussus abietinus	Oabi002361.1<br>Oabi004554.1	Oabi002436.1	Oabi002175.1	Oabi007309.1	Oabi006338.1	Oabi008238.1	Oabi002350.1<br>Oabi008225.1	Oabi006967.1
Pteromalus puparum	Ppup014352.1<br>Ppup015083.1	Ppup002696.1	Ppup001426.1	Ppup009704.1<br>Ppup009706.1	Ppup014683.1	Ppup001157.1	Ppup002689.1<br>Ppup015056.1	Ppup005996.1
Pachycrepoideus vindemmiae	OQ145164	OQ145166	OQ145167	OQ145168	OQ145169	OQ145170	OQ145171	OQ145172
Sycophaga agraensis	Sagr019502.1	Sagr006235.1	Sagr006456.1	Sagr009125.1<br>Sagr009138.1	Sagr012107.1<br>Sagr012105.1	Sagr013413.1	Sagr000858.1<br>Sagr022112.1	Sagr005464.1
Synergus gifuensis	Sgif017749.1<br>Sgif020467.1	Sgif010233.1	Sgif017653.1	Sgif020843.1<br>Sgif020848.1	Sgif026810.1	Sgif004117.1	Sgif004472.1<br>Sgif012416.1	Sgif013540.1
Synergus itoensis	Sito008686.1<br>Sito009213.1	Sito017573.1	Sito016000.1	Sito016790.1<br>Sito016795.1	Sito002351.1	Sito006926.1	Sito012727.1<br>Sito019065.1	Sito026406.1
Trichogramma brassicae	Tbra001487.1	Tbra_CAB0037618.1	Tbra001021.1	Tbra014209.1	Tbra014358.1	Tbra010113.1	Tbra015453.1	Tbra002083.1
Trichogramma evanescens	Teva000475.1	Teva001526.1	Teva013308.1	Teva002620.1<br>Teva018761.1	Teva007200.1<br>Teva009884.1	Teva013514.1	Teva003303.1	Teva004685.1
Trichogramma pretiosum	Tpre003920.1		Tpre010313.1	Tpre006762.1	Tpre_XP_023314962.1<br>Tpre_XP_014227574.1<br>Tpre_XP_023314964.1	Tpre010009.1	Tpre011434.1	Tpre009158.1
Trichomalopsis sarcophagae	Tsar010023.1	Tsar008034.1	Tsar012702.1	Tsar011862.1	Tsar004315.1		Tsar006819.1	Tsar006116.1

	Tsar014165.1			Tsar015339.1			Tsar007251.1	
Venturia canescens	Vcan014526.1	Vcan003998.1	Vcan004165.1	Vcan019668.1	Vcan019859.1	Vcan010183.1	Vcan002730.1	Vcan018314.1
				Vcan019675.1			Vcan021591.1	
