# Supplementary material for: Circadian Activity and Clock Genes in Pachycrepoideus vindemmiae: Implications for Field Applications and Circadian Clock Mechanisms of Parasitoid Wasps
Source: Insects. 2023 May 22;14(5):486. doi: 10.3390/insects14050486 (PMC10231066; doi:10.3390/insects14050486)
Supplement: Supplementary file 1 [file insects-14-00486-s001.zip › Table S2.pdf]

Table S2. Primers used in qPCR analysis

Primer name	Primer（5'-3'）
Pv_Clk-SP	CAGTCTACCGAGATGCGTCA
Pv_Clk-AP	CGTCGTTTGCTGACATGAGT
Pv_cyc-SP	CCCGACAACCAAGAGTTCAT
Pv_cyc-AP	CCGAAGTACACTGTGGAGCA
Pv_per-SP	GCTACGGCCAGCTCAACTAC
Pv_per-AP	ATTCTCAGCACTGCCGCTAT
Pv_timeout-SP	TGCCAGTGACCAAGATACCA
Pv_timeout-AP	TGCAAGCCTAGCAGGATTTT
Pv_cry2-SP	CAGACCGGATTGTCTCCCTA
Pv_cry2-AP	GACGTTCTTGTCCCAGGGTA
Pv_vrille-SP	GGCTTCCTTTACCAGGGAAC
Pv_vrille-AP	TTCTCCTTGCTGAGCTCCAT
Pv_Pdp1-SP	GTTCGTACCCGACGACCTAA
Pv_Pdp1-AP	CCGTGTACTTGCTCAGCTTG
Pv_cwo-SP	TCCAGCTCATCAGTCACCTG
Pv_cwo-AP	GTGACTGCTGTGGTTGCTGT
